# Postbiotics and paraprobiotics in food biochemistry mechanisms stability and nutritional applications

**DOI:** 10.1038/s41538-026-00838-z

**Published:** 2026-04-16

**Authors:** Said Dahmouni, Djilali Benabdelmoumene, Zineb Bengharbi, Mustapha Kamel Fodil, Kabir Mustapha Umar, Wasim S. M. Qadi, Farial Krache, Muhammad Waqar, Xiaohui Tong, Rongchun Han, Zulzikry Hafiz Abu Bakar, Murni Nazira Sarian, Ahmed Mediani, Faidruz Azura Jam, Hamizah Shahirah Hamezah

**Affiliations:** 1 Applied Animal Physiology Lab, Abdelhamid Ibn Badis University, Mostaganem, Algeria; 2 Centre for Dryland Agriculture, Bayero University, Kano, Nigeria; 3 Institute of Systems Biology (INBIOSIS), Universiti Kebangsaan Malaysia, Bangi, Malaysia; 4 Food Technology and Innovation Research Center of Excellence, School of Agricultural Technology and Food Industry, Walailak University, Nakhon Si Thammarat, Thailand; 5 School of Life Sciences, Anhui University of Chinese Medicine, Hefei, China; 6 School of Pharmacy, Anhui University of Chinese Medicine, Hefei, China; 7 Centre for Defence Foundation Studies, National Defence University of Malaysia, Sungai Besi Camp, Kuala Lumpur, Malaysia; 8 Department of Biochemistry, Faculty of Medicine, Manipal University College Malaysia (MUCM), Melaka, Malaysia

**Keywords:** Biotechnology, Computational biology and bioinformatics, Microbiology

## Abstract

Postbiotics, defined as preparations of inanimate microorganisms and their components that confer health benefits, are emerging as stable and safe alternatives to probiotics. This review summarizes recent advances in the biochemical composition, mechanisms of action, and applications of postbiotics and paraprobiotics in human and animal nutrition. It also highlights innovations in omics technologies, artificial intelligence, and sustainable production strategies that are shaping next-generation microbiome-based functional foods and therapeutic interventions.

## Introduction

Over the past two decades, advances in gut microbiome research have profoundly reshaped food science, positioning diet–microbiota–host interactions at the forefront of nutritional innovation. Probiotics, classically defined as live microorganisms that confer health benefits when administered in adequate amounts, have been central to this transformation^1,2^. However, growing evidence has revealed several limitations associated with probiotic use, including reduced viability, instability during processing and storage, interindividual variability in colonization, and potential safety concerns in vulnerable populations, prompting the search for alternative microbiome-derived strategies^3,4^.

Postbiotics represent a conceptual and technological transition from live microbial supplementation to molecule- and structure- driven bioactivity. The 2021 consensus definition issued by the International Scientific Association for Probiotics and Prebiotics (ISAPP) formalized postbiotics as “preparations of inanimate microorganisms and/or their components that confer a health benefit on the host, thereby decoupling efficacy from microbial viability^2^. In parallel, paraprobiotics have gained attention as biologically active entities despite lacking metabolic activity^3,5^. Collectively, these concepts reflect a paradigm shift in food biochemistry in which probiotic effects are increasingly attributed to microbial metabolites and structural components rather than to microbial colonization per se^6^. From a biochemical perspective, postbiotics encompass a diverse repertoire of microbially-derived compounds, including short-chain fatty acids (SCFAs), organic acids, antimicrobial peptides, enzymes, exopolysaccharides (EPS), and cell wall constituents such as peptidoglycans and teichoic acids (TAs)^7^. These molecules interact with host signaling networks through multiple mechanisms, including activation of G-protein-coupled receptors, modulation of epithelial barrier integrity, and engagement of pattern recognition receptors on immune cells, thereby influencing inflammation, immune homeostasis, and metabolic regulation^8,9^. Beyond their mechanistic relevance, postbiotics and paraprobiotics offer important technological advantages for food systems. Their intrinsic stability under thermal, acidic, and oxidative conditions facilitates their incorporation into a wide range of food matrices without reliance on cold chains or viability guarantees, while their non-viable nature improves safety profiles for applications in infant nutrition, clinical settings, and immunocompromised populations^3,10^. The translational significance of postbiotics extends across both human and animal nutrition. In humans, postbiotic-enriched foods and supplements are increasingly investigated for supporting gut health, modulating immune responses, and improving metabolic function^6,10^.

In animal production systems, including poultry, livestock, and aquaculture, postbiotics have emerged as promising alternatives to antibiotic growth promoters, with reported improvements in gut morphology, immune competence, feed efficiency, and disease resistance^11,12^. Despite the rapid growth of the literature, the postbiotic field remains characterized by conceptual fragmentation. Inconsistencies in terminology, heterogeneity in production and analytical methods, and limited standardization of dose metrics continue to hinder cross-study comparability and regulatory harmonization^13^. Moreover, mechanistic resolution often lags behind empirical observations, reflecting the complexity of postbiotic compositions and their multi-target modes of action. Recent advances in metabolomics, proteomics, systems biology, and AI provide unprecedented opportunities to overcome these limitations. Integrated omics pipelines enable high-resolution characterization of postbiotic preparations, while network biology and machine learning (ML) models support predictive mapping of structure–function relationships and biomarker-driven personalization^14,15^.

In parallel, the valorization of agro-industrial by-products as fermentation substrates positions postbiotics within circular bioeconomy frameworks, aligning functional food innovation with sustainability imperatives^16^. Although several reviews have addressed postbiotics from nutritional, microbiological, or clinical perspectives, few have systematically integrated molecular mediators, mechanistic pathways, technological stability, and emerging computational approaches across both human and animal nutrition^16–18^. The present review addresses this gap by synthesizing evidence from 2020 to 2026 to provide a unified food- biochemistry perspective on postbiotics and paraprobiotics, outlining future directions for research, industrial innovation, and regulatory alignment.

## Review methodology

This narrative review synthesizes peer-reviewed literature published between January 2020 and March 2026, focusing on postbiotics and paraprobiotics in the context of food biochemistry, human nutrition, and animal nutrition. Literature was identified through targeted searches of the PubMed, Scopus, and Web of Science using combinations of keywords including “postbiotics*“*, “paraprobiotics“, “inactivated probiotics“, “microbial metabolites“, and “non-viable microorganisms“. Both original research articles and authoritative reviews were considered. Priority was given to studies that provided clear characterization of postbiotic or paraprobiotic preparations and reported measurable biological, physiological, or technological outcomes relevant to food systems. Regulatory documents and consensus statements were included where appropriate to contextualize definitions and emerging standards. The resulting body of literature was critically evaluated and integrated to identify areas of consensus, mechanistic insights, and remaining research gaps.

## Definitions and taxonomy

### Consensus definitions (ISAPP 2021)

Over the past decade, the rapid expansion of microbiome-based interventions has necessitated the establishment of standardized terminology to ensure scientific consistency and regulatory clarity. Probiotics are defined as “live microorganisms that, when administered in adequate amounts, confer a health benefit on the host”^1^, while prebiotics are “substrates selectively utilized by host microorganisms, conferring a health benefit”^1^. Building on these foundational concepts, synbiotics were formally defined as *“*a mixture comprising live microorganisms and substrate(s) selectively utilized by host microorganisms, that confers a health benefit on the host”^19^.

Importantly, synbiotics may be either complementary, in which probiotic and prebiotic act independently, or synergistic, in which the substrate is specifically designed to support the co-administered microorganism, reflecting an evolution toward functionally targeted microbiome modulation. Together, these consensus definitions establish clear criteria for classical biotic interventions and provided a conceptual framework for emerging microbiome-derived strategies (Table 1). The most consequential development for the present review is the standardization of the postbiotic concept by the ISAPP. Following an expert consultation initiated in 2019, ISAPP defined a postbiotic as “a preparation of inanimate microorganisms and/or their components that confers a health benefit on the host”^2^. This definition represents a decisive conceptual shift, formally decoupling health efficacy from microbial viability. The deliberate use of the term *“*inanimate*”*, rather than *“*inactive*”*, emphasizes that microbial cells are rendered non-living while potentially retaining biologically active structural and molecular features^20^.

**Table 1:** Comparative overview of major “-biotic” classes, definitions, examples, and key distinguishing features

Category	Accepted Definition	Representative Microbes or Substrates	Mechanistic Basis	Key Distinguishing Features	Consensus Framework	References
**Probiotics**	Live microorganisms that, when administered in adequate amounts, confer a health benefit on the host	*Lactobacillus, Bifidobacterium, S. boulardii*	Colonization, competitive exclusion of pathogens, metabolite production (SCFAs, vitamins), immune modulation	Viability essential; require survival through manufacturing and GI transit	FAO/WHO Guidelines (2002); ISAPP consensus definition	^1,22^
**Prebiotics**	Substrates that are selectively utilized by host microorganisms conferring a health benefit	Inulin, fructo-oligosaccharides (FOS), galacto-oligosaccharides (GOS), resistant starches	Selective fermentation stimulating beneficial microbiota; production of SCFAs	Non-viable dietary components; act as “food” for microbes	ISAPP consensus definition	^19^
**Synbiotics**	Mixture comprising live microorganisms and substrate(s) selectively utilized by host microorganisms that confers a health benefit on the host	Probiotic + prebiotic pairings (*Bifidobacterium*+ inulin)	Complementary or synergistic actions on gut microbiota and host metabolism	Requires both viable microbes and compatible substrate; two design types: synergistic or complementary	ISAPP consensus statement	^19,29^
**Paraprobiotics**	Non-viable microbial cells (intact or broken) or cell fractions which, when administered in adequate amounts, confer a benefit on the consumer	Heat-killed *L. rhamnosus, B. coagulans, Enterococcus faecalis*	Interaction of inactivated cell structures (PG, LTA, surface proteins) with host PRRs → immunomodulation	Viability not required; safety advantage for immunocompromised populations	Concept proposed in scientific literature	^5,25,208^
**Postbiotics**	Preparations of inanimate microorganisms and/or their components that confer a health benefit on the host	Cell-free supernatants, SCFAs, peptides, EPS, muramyl peptides	Direct biochemical signaling via metabolites and microbial molecules → immune, barrier, and metabolic regulation	Broadest category; includes both inactivated cells and soluble metabolites; inherently stable and safe	Concept proposed in scientific literature	^2,22,92^
**Psychobiotics / Functional Modifiers**	Microbiome-targeted or derived interventions that modulate brain and behavior via the gut–brain axis	*L.rhamnosus* GG, *B. longum* 1714, tryptophan-derived metabolites	Production of neuroactive compounds (GABA, 5-HT precursors, SCFAs); vagal and immune signaling	Emerging subclass; overlaps with probiotic/postbiotic domains; still lacks regulatory definition	Concept proposed in scientific literature	^31,209^

Equally critical, the term *“*preparation*”* underscores that postbiotics are formulated end-products whose biological activity depends on strain identity, growth conditions, matrix composition, and inactivation method^2,20^. A defining requirement of the ISAPP consensus is that postbiotic preparations must demonstrate a measurable health benefit in the target host, aligning postbiotics with the evidentiary standards applied to probiotics^10^. This criterion excludes loosely defined microbial metabolites or fermentation by-products lacking demonstrated bioactivity, a distinction that has been critical in addressing widespread terminological misuse in both scientific literature and commercial claims. Subsequent authoritative reviews and position papers have reinforced the ISAPP definition as the prevailing reference framework in food, nutrition, and pharmaceutical sciences, while clarifying common misconceptions regarding postbiotic composition, characterization, and scope^21,22^. As of 2025, the ISAPP definition remains the cornerstone taxonomy for postbiotics, with increasing acknowledgment by regulatory bodies and standard-setting organizations worldwide^13^. While debate persists regarding the boundaries between postbiotics, paraprobiotics, and isolated microbial metabolites, broad consensus now exists that rigorous characterization and demonstrated host benefit are indispensable criteria. This standardized framework provides a critical foundation for advancing mechanistic research, regulatory alignment, and the rational development of microbiome-derived functional foods.

### Postbiotics vs. paraprobiotics: similarities and differences

The term “paraprobiotic“ predates the formalization of the postbiotic concept and was introduced to describe non-viable microbial cells that retain health-promoting properties. Taverniti and Guglielmetti^5^ originally defined paraprobiotics as “non-viable microbial cells (intact or broken) or crude cell extracts which, when administered in adequate amounts, confer a benefit on the consumer”. This definition was motivated by early observations that microbial viability is not a prerequisite for biological activity, particularly with respect to immunomodulation and host–microbe signaling. Conceptually, paraprobiotics and postbiotics share a common rationale: the intentional use of inactivated microorganisms to deliver health benefits while overcoming limitations associated with live probiotics. Both approaches are increasingly valued for their enhanced safety profile and technological robustness. Because non-viable microbial cells lack replicative capacity, they are unable to proliferate in the host, which markedly reduces risks related to uncontrolled colonization, epithelial translocation, and the horizontal transfer of antibiotic resistance determinants. Moreover, inactivated cells and their derived bioactive fractions generally exhibit improved stability during industrial processing and storage, supporting more consistent functional performance across formulations and supply chains^23–25^.

Experimental and clinical studies consistently demonstrate that heat-killed or otherwise inactivated bacteria can modulate immune responses, reduce infection risk, and exert metabolic effects comparable to those of live probiotics, but without the associated safety concerns^25^. Despite these shared foundations, key differences in scope and formal definition distinguish paraprobiotics from postbiotics. The original paraprobiotic concept focused narrowly on non-viable microbial cells and cell fragments, without explicitly accounting for microbial metabolites that may also be present in the preparation^5^. In contrast, the ISAPP postbiotic definition deliberately adopts a broader and more inclusive framework, encompassing *“*preparations of inanimate microorganisms and/or their components “, with or without associated metabolites, provided that a health benefit is demonstrated in the target host^2,22^.

**Fig. 1:**
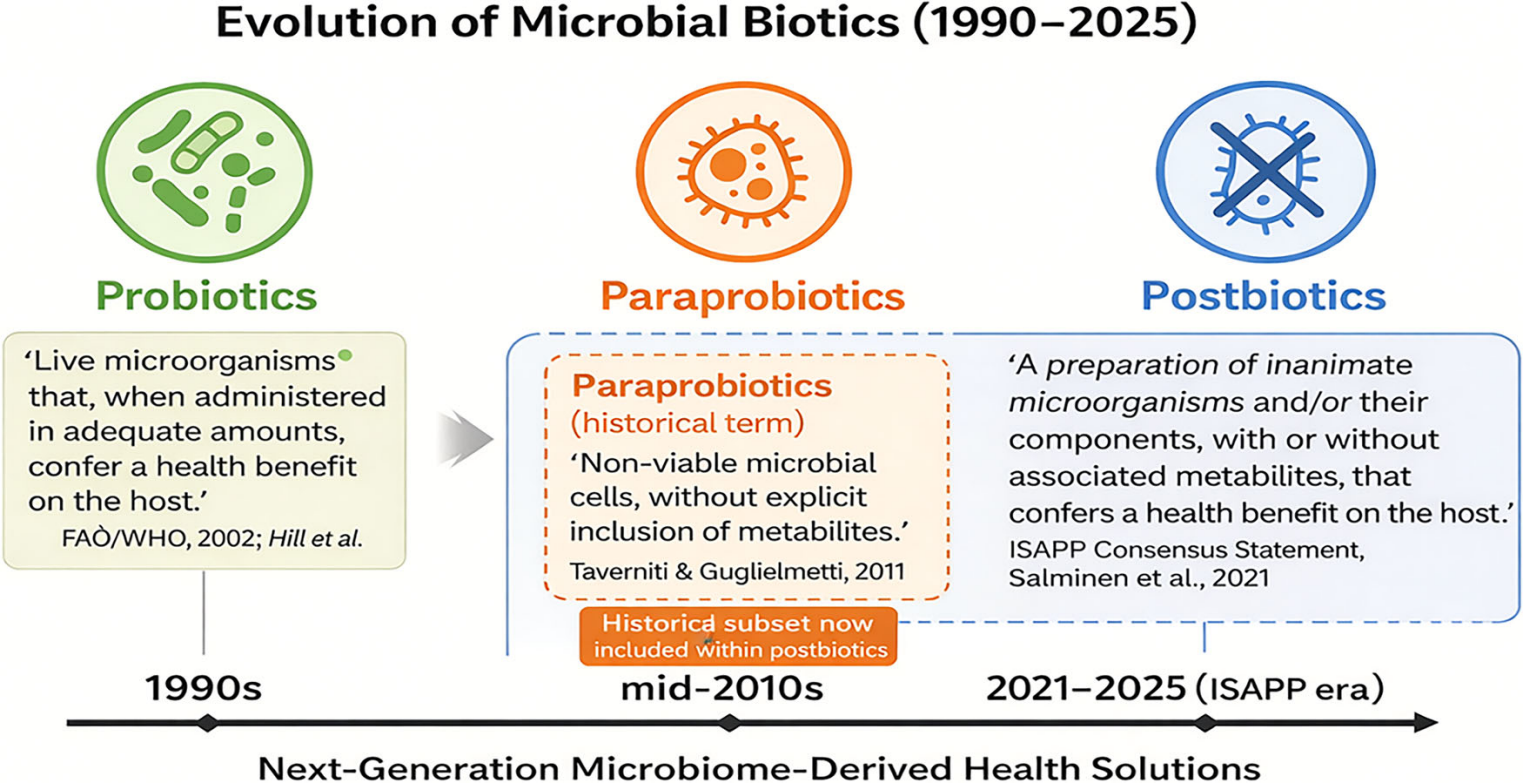
Harmonized taxonomy and conceptual evolution of microbiome-derived “biotic” interventions. The diagram outlines the historical progression of microbial health definitions from the 1990s to 2025. Probiotics (left) and paraprobiotics (center) transition into the contemporary ISAPP definition of postbiotics (right). The dashed box denotes that paraprobiotics are now considered a historical subset subsumed under postbiotics. The bottom solid arrow indicates the overall chronological timeline.

This distinction is particularly relevant in practical applications, as most inactivated microbial preparations inherently contain a combination of structural cell components and fermentation-derived metabolites. Recent clinical evidence further supports this integrative perspective. For instance, Mutoh et al.^26^ reported that heat-killed *Lactobacillus helveticus* improved mood states in healthy adults, while Komatsu et al.^27^ observed comparable functional benefits using heat-killed lactic acid bacteria (LAB) in a randomized controlled trial. These findings confirm that microbial inactivation does not abolish functional properties, reinforcing the notion that biological activity is primarily mediated by microbial structures and molecular signals rather than cellular viability alone^28^. Taken together, current evidence supports the view that paraprobiotics, as originally conceived, are best regarded as a historical and operational subset within the broader postbiotic framework. The ISAPP definition provides a more rigorous, standardized, and regulatory-relevant taxonomy by explicitly requiring product characterization and demonstrated host benefit, while accommodating the biochemical complexity of inactivated microbial preparations (Fig. 1). Consequently, although the term “paraprobiotic” remains useful in describing specific inactivation-based strategies, its functional and conceptual scope is now largely encompassed by the postbiotic paradigm^25^. This clarification is essential for harmonizing terminology, guiding product development, and ensuring consistency in scientific communication and regulatory evaluation.

### Synbiotics, psychobiotics, and other related terms

Beyond probiotics, prebiotics, and postbiotics, the lexicon of microbiome science has expanded to include several additional “-biotic” terms intended to capture specific functional strategies. Among these, synbiotics represent the only category, aside from postbiotics, that has been formally standardized through international consensus. Synbiotics refer to products combining live microorganisms with substrates selectively utilized by host microorganisms in either a synergistic or complementary manner. Although the concept originated in the 1990s, a consensus panel in 2020 refined the definition to emphasize demonstrated health benefits and functional intent^19^. In synergistic synbiotics, the substrate is specifically designed to support the co-administered microorganism, whereas complementary synbiotics combine independently beneficial probiotics and prebiotics. Recent studies increasingly focus on the rational design of synbiotic, leveraging strain–substrate specificity to enhance functional synergy and reproducibility^29,30^.

In contrast, psychobiotics represent a functionally motivated but non-consensus term. Originally introduced by Dinan et al.^31^, psychobiotics were defined as live bacteria that, when ingested in adequate amounts, exert anxiolytic or antidepressant effects through microbiome–gut–brain axis interactions. Subsequent usage has broadened the term to include prebiotics and dietary interventions capable of modulating neurobehavioral outcomes via microbial pathways^32^. Contemporary reviews highlight rapid growth in psychobiotics research, with increasing emphasis on precision nutrition, responder stratification, and neuroimmune signaling^33–35^. However, despite its conceptual appeal, the term psychobiotics lacks a formal consensus definition, regulatory recognition, and standardized criteria for product characterization, limiting its taxonomic robustness. Additional neologisms have been proposed to emphasize specific functional domains such as immune modulation, cancer therapy, or pharmaceutical applications of microbes. While these terms may be useful as descriptive shorthand within specialized contexts, their proliferation risks conceptual dilution and regulatory ambiguity. Consequently, expert consensus increasingly favors restricting formal taxonomy to rigorously defined categories while describing specific functional outcomes within this established framework^2,22^. Accordingly, synbiotics remain the only additional “-biotic” category supported by international consensus, whereas psychobiotics and related neologisms are best regarded as functional descriptors rather than distinct taxonomic entities. This distinction is essential for maintaining terminological clarity, ensuring regulatory coherence, and preventing the unchecked expansion of microbiome nomenclature in both scientific literature and commercial practice.

### Historical confusion in terminology and current clarifications

The rapid expansion of microbiome-related products has historically been accompanied by substantial terminological inconsistency. Prior to the 2021 ISAPP consensus, a wide array of terms was frequently used interchangeably to describe non-viable microbial preparations^10^. This confusion was further compounded by inconsistent definitions of postbiotics, with some authors restricting the term to cell-free metabolites and others applying it broadly to any inactivated microbial product. Consequently, identical or highly similar preparations were often classified under different labels across studies, severely limiting cross-study comparability and the synthesis of evidence. The ISAPP consensus statement published in 2021 marked a critical inflection point by unifying this fragmented landscape under a single, rigorously defined postbiotic framework^2^.

**Fig. 2:**
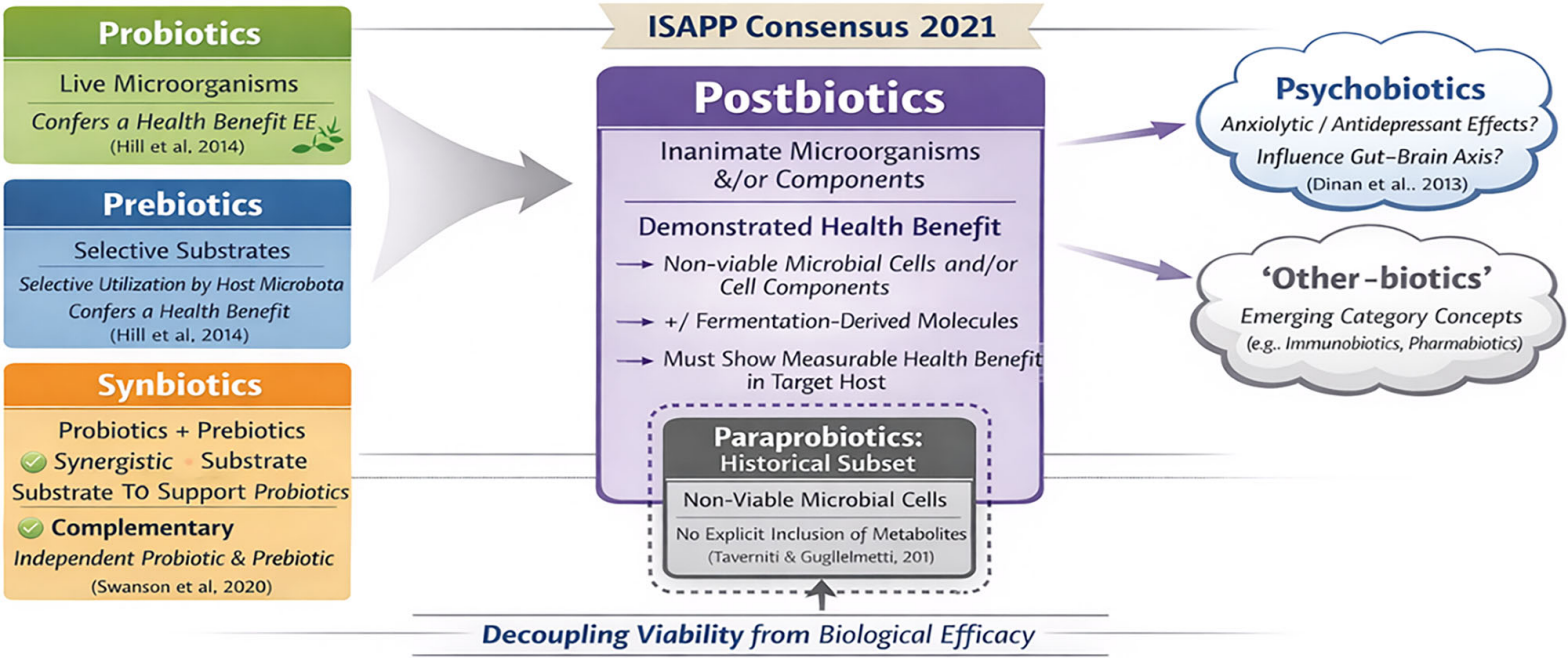
Conceptual framework of probiotics, prebiotics, synbiotics, and postbiotics based on the ISAPP consensus definition. The diagram maps the transition from traditional interventions (probiotics, prebiotics, and synbiotics, left) to the modern postbiotics category (center), highlighting the decoupling of microbial viability from biological efficacy. A central dashed box designates paraprobiotics as a historical subset subsumed by postbiotics. Arrows pointing tothe right indicate the expansion of these frameworks into specialized, emerging categories such as psychobiotics and other descriptive biotics.

Subsequent bibliometric analyses demonstrate that the term “postbiotics“ has rapidly emerged as the dominant keyword in the field, progressively displacing older and less precisely defined terms such as paraprobiotic*s* and bacterial lysates^36,37^. Although debate persists regarding the precise boundaries of postbiotic composition, the ISAPP definition is now widely cited as the authoritative reference across food, nutrition, and pharmaceutical sciences^13^. Importantly, this standardization has also exposed persistent challenges. Recent commentaries and preprints have highlighted substantial heterogeneity in the scientific rigor and expertise reflected in postbiotic review articles published since 2021, with some failing to adhere to the ISAPP criteria or conflating distinct categories of microbiome-derived products^13^. These observations underscore the need for continued vigilance in terminology use, particularly as commercial interest accelerates and new applications emerge. Overall, however, terminology in the postbiotic field is substantially more coherent in 2025 than in the preceding decade, enabling more reliable cross-study comparison, mechanistic synthesis, and regulatory interpretation. The consolidation of nomenclature around the ISAPP framework has therefore been instrumental in advancing conceptual clarity and supporting the maturation of postbiotics as a scientifically and industrially credible category (Fig. 2).

In addition to defined microbiome-derived interventions such as probiotics, prebiotics, and postbiotics, fecal microbiota transplantation (FMT) has emerged as another strategy for restoring microbial balance in the gut ecosystem. FMT involves the transfer of processed fecal material from a healthy donor to a recipient in order to re-establish a diverse and functional intestinal microbiota^38^. This approach has demonstrated high clinical efficacy in the treatment of recurrent *Clostridioides difficile* infection and is increasingly being investigated for other dysbiosis-related disorders. Unlike postbiotics or paraprobiotics, which rely on defined microbial preparations or bioactive metabolites, FMT introduces a complex microbial community, which presents both therapeutic opportunities and challenges related to safety, standardization, and regulatory oversight^39^.

### Proposed classification framework

**Fig. 3:**
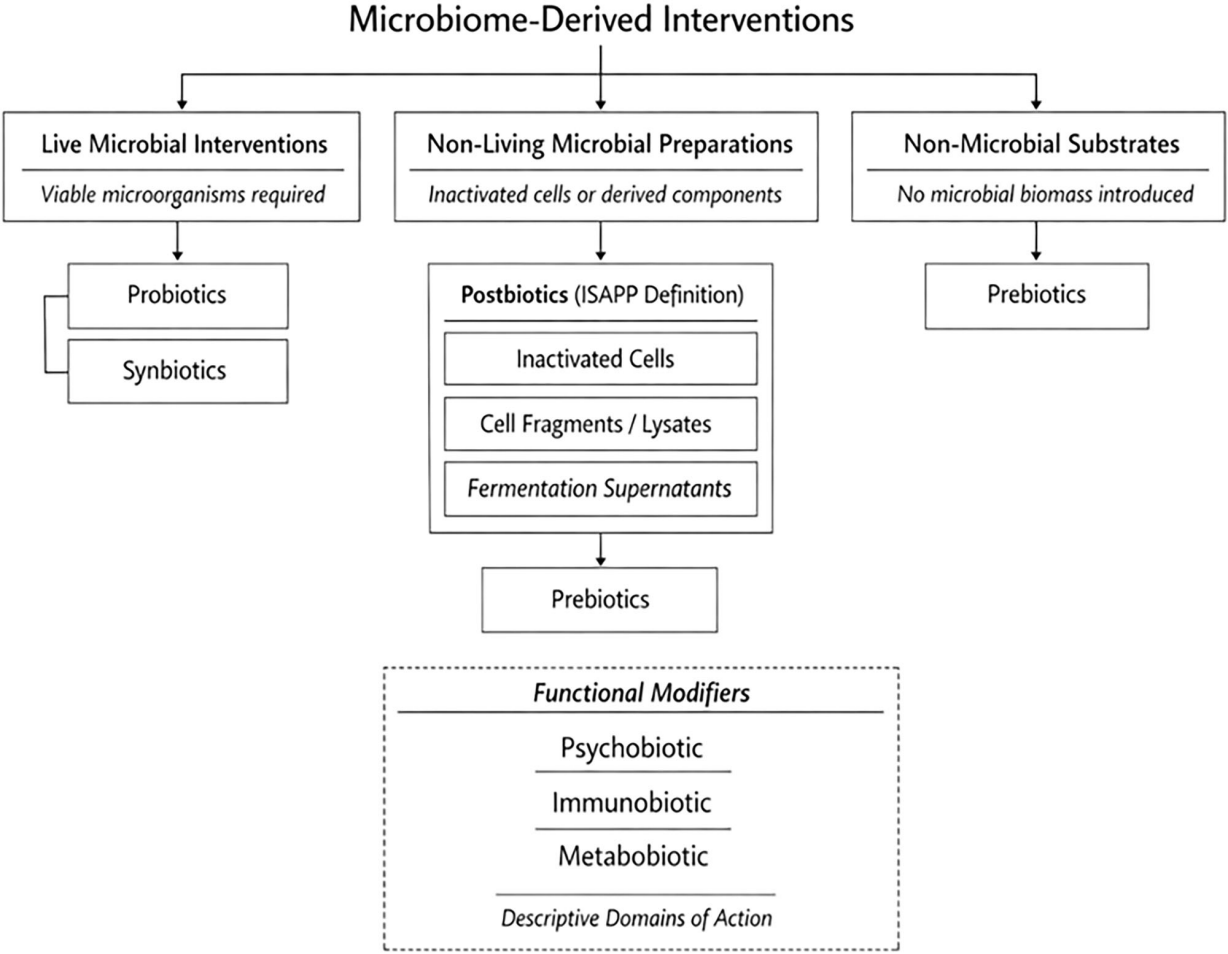
Taxonomic relationships among microbial biotics. The flowchart categorizes microbiome-derived interventions into three primary branches: live microbial interventions, non-living microbial preparations (postbiotics), and non-microbial substrates (prebiotics). Solid arrows denote hierarchical classification. The bottom dashed box highlights functional modifiers (psychobiotic, immunobiotic, metabobiotic) that serve as descriptive domains of action.

Building on recent consensus definitions and efforts to harmonize microbiome-related terminology, we propose a structured and operational classification framework for microbiome-derived interventions (Fig. 3). At the highest level, interventions can be distinguished according to microbial viability and functional role into three non-overlapping categories: live microbial interventions, non-living microbial preparations, and non-microbial substrates. Live microbial interventions comprise probiotics and synbiotics, for which biological activity depends on the presence of viable microorganisms. Non-living microbial preparations are encompassed within the postbiotic category as defined by the ISAPP consensus, including inactivated whole cells, cell fragments or lysates, and fermentation-derived supernatants, provided that a measurable health benefit is demonstrated in the target host. Non-microbial substrates are represented by prebiotics, which selectively modulate host microbiota activity without introducing microbial biomass. Within this unified framework, function-oriented terms such as “psychobiotic” or “immunobiotic” are best regarded as descriptive modifiers rather than independent taxonomic categories, specifying the physiological domain of action within an established class^2,20^. Recent perspectives further emphasize the importance of quantitative characterization and standardization of postbiotic preparations to support regulatory clarity and reproducibility^40^, alongside increasing convergence between the food and pharmaceutical sectors that necessitates harmonized commercial and regulatory approaches^41^.

Early regulatory guidance emerging from Australia, China, and Europe increasingly endorses the adoption of a broad, umbrella definition of postbiotics consistent with the ISAPP framework^13^. Collectively, this classification reduces terminological redundancy, enhances conceptual clarity, and aligns scientific communication with international consensus definitions, providing a robust and future-oriented taxonomy to guide research, product development, and regulatory evaluation. Table 1 summarizes the defining criteria, representative examples, and mechanistic distinctions among probiotic, prebiotic, synbiotic, paraprobiotic, and postbiotic categories, while Fig. 3 illustrates the hierarchical taxonomy of microbiome-derived “biotic” interventions, distinguishing live, inactivated, and metabolite-associated approaches within a coherent conceptual structure.

## Biochemistry of postbiotic molecules

**Fig. 4:**
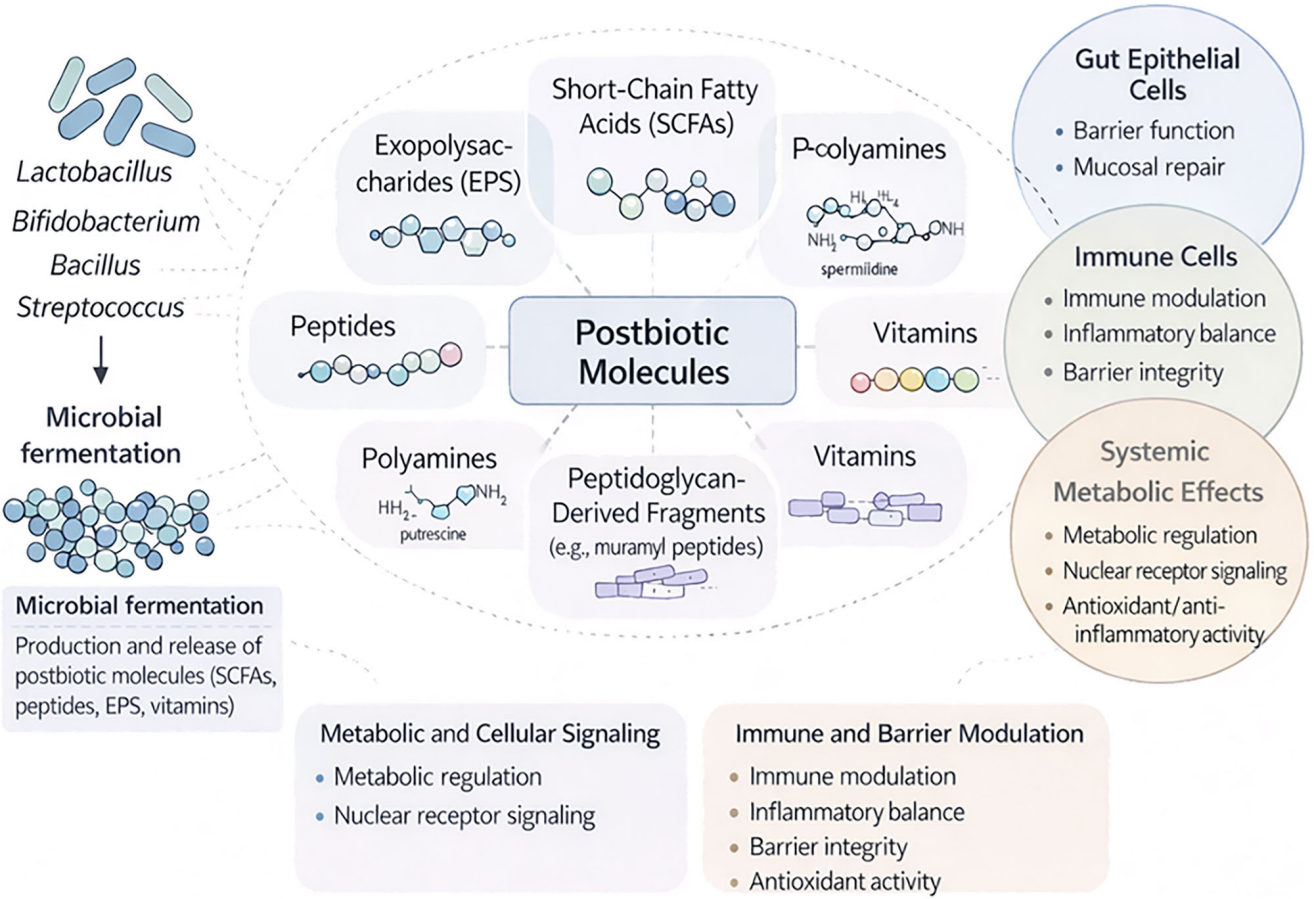
Overview of major classes of postbiotic molecules produced by microbial fermentation and their principal host targets. The diagram illustrates bacterial strains undergoing fermentation to produce postbiotic molecules (SCFAs, EPS, peptides, polyamines, peptidoglycan fragments, and vitamins). These molecules target gut epithelial cells, immune cells, and systemic pathways to modulate barrier function, inflammatory balance, and metabolic regulation.

Postbiotic molecules encompass a diverse array of biochemical entities derived from probiotic or commensal microorganisms, including metabolic byproducts such as SCFAs and polyamines, structural cell components such as peptidoglycan fragments and lipoteichoic acids (LTAs), secreted polymers such as EPS, and bioactive proteins or peptides. These molecules mediate host effects through defined biochemical pathways, including receptor-mediated immune signaling, modulation of epithelial barrier function, and metabolic regulation. As summarized in Table 2, postbiotic compounds span organic acids, peptides, polysaccharides, and polyamines, each characterized by distinct microbial origins and specific mechanistic modes of action. Figure 4 integrates the principal metabolic routes underlying postbiotic production with their corresponding host targets and physiological outcomes.

**Table 2:** Biochemical diversity of postbiotic molecules: microbial origins, mechanisms, and physiological effects

Class of molecule	Representative sources/producers	Primary biochemical mechanism	Physiological or functional effect	Notes/key insights	References
Short-chain fatty acids (SCFAs) (acetate, propionate, butyrate)	*Lactobacillus*, *Bifidobacterium, Clostridium, Faecalibacterium*	Production through microbial fermentation of carbohydrates and fibers; activation of G-protein-coupled receptors (GPR41 and GPR43) and modulation of histone acetylation	Enhancement of intestinal barrier integrity, regulation of glucose and lipid metabolism, anti-inflammatory activity, and neuroactive effects	Central metabolic mediators linking gut microbiota to host energy homeostasis and immune regulation	^127,210,211^
Organic acids (lactic, citric, formic acids)	*Lactobacillus, Pediococcus, Leuconostoc*	Reduction of environmental pH and alteration of pathogen membrane permeability	Antimicrobial preservation and suppression of pathogenic microorganisms	Key contributors to microbial safety and stability in fermented foods	^92^
Peptidoglycan fragments / MDP	Gram-positive *Lactobacillus, Bacillus*	Recognition by intracellular PRRs such as NOD2, associated with modulation of NF-κB and MAPK signaling pathways	Controlled immune priming and promotion of immune tolerance	Structural molecular signals characteristic of inactivated microbial cells	^22,212^
Lipoteichoic acids (LTAs)	*L. casei, L. plantarum*	Interaction with TLR2, resulting in context-dependent cytokine modulation	Regulation of pro- and anti-inflammatory immune responses	Immunomodulatory effects are dose- and structure-dependent	^213,214^
Exopolysaccharides (EPS)	*L. rhamnosus* GG*, B. longum, Streptococcus thermophilus*	Modulation of immune signaling pathways, antioxidant activity, and contribution to biofilm matrix formation	Antioxidant, anti-allergic, and cholesterol-lowering effects	Strain-specific bioactivity with relevance to functional food formulation	^65,215^
Bacteriocins and bioactive peptides (nisin, pediocin, p40/p75)	*L. lactis, E. faecalis, L. rhamnosus* GG	Antimicrobial pore-forming activity and association with modulation of host epithelial signaling pathways such as ERK and Akt	Inhibition of pathogens, epithelial protection, and anti-inflammatory effects	Can exert both local intestinal and systemic effects	^2,44^
Cell wall–derived ligands (TAs, lipopolysaccharide derivatives)	*Bacillus, L. plantarum, Escherichia coli Nissle* 1917	Activation of PRRs (TLR2 and TLR4) associated with immune signaling modulation	Induction of mucosal tolerance and regulation of cytokine cascades	Distinct signaling properties between Gram-positive and Gram-negative derivatives	^67^
Heat-shock proteins and antioxidant enzymes (HSPs, SOD, CAT)	Inactivated *Lactobacillus* and *Bifidobacterium*	Stimulation of epithelial stress-response pathways and regulation of cellular redox balance	Cytoprotection and reduction of oxidative damage	Emerging postbiotic components in stress adaptation and resilience	^69,216^
Vitamins and cofactors (B-group vitamins, vitamin K2)	*Lactococcus, Propionibacterium, Bifidobacterium*	Contribution to host metabolic pathways as enzymatic cofactors and antioxidants	Nutritional supplementation, bone health, and metabolic support	Frequently co-produced alongside SCFAs in fermentation processes	^217^
Polyamines (putrescine, spermidine, spermine)	*L. plantarum, E. faecalis*	Regulation of DNA stabilization, oxidative stress responses, and autophagy-related signaling	Promotion of gut maturation, epithelial repair, and anti-aging effects	Increasing relevance in gut–brain axis research	^44,72^
Quorum-sensing peptides and autoinducers	Multiple LAB and *Bacillus* species	Modulation of microbial communication and host gene expression	Interference with pathogenic colonization and microbial cross-talk	Interface between microbial ecology and host epithelial responses	^11^

### Microbial-derived metabolites

#### Short-chain fatty acids (SCFAs)

Short-chain fatty acids (SCFAs), primarily acetate, propionate, and butyrate, are generated through microbial fermentation of dietary fibers in the colon and typically occur in an approximate molar ratio of 60:20:20 in healthy individuals^42,43^. Alterations in this profile have been consistently associated with impaired gut homeostasis and metabolic dysregulation^44^. Beyond serving as energy substrates, SCFAs act as key signaling molecules that influence host metabolism and immunity. They enhance insulin sensitivity and regulate lipid and glucose metabolism through activation of nuclear receptors such as peroxisome proliferator-activated receptor gamma (PPARγ) and downstream metabolic pathways. In parallel, SCFAs engage G protein–coupled receptors, including GPR41 and GPR43, expressed on immune and epithelial cells, thereby promoting regulatory T -cell differentiation and suppressing pro-inflammatory responses. Among SCFAs, butyrate plays a particularly critical role in maintaining intestinal barrier integrity by serving as the primary energy source for colonocytes and by upregulating tight junction proteins, thereby limiting endotoxin translocation and low-grade inflammation^44^.

#### Organic acids

In addition to SCFAs, gut microbes produce a range of organic acids, including lactic, succinic, and formic acids, which exert both ecological and host-directed effects^45^. Lactic acid, predominantly produced by LAB, lowers luminal pH, creating an environment that favors beneficial taxa such as Lactobacillus and Bifidobacterium while inhibiting acid-sensitive pathogens, including members of the Enterobacteriaceae family^46^. Beyond pH modulation, organic acids can function as bioactive signaling molecules. For instance, luminal lactic acid has been shown to stimulate intestinal dendritic cells through GPR31-dependent pathways, thereby enhancing mucosal immune surveillance and antimicrobial defense^44^. Through the combined effects of pathogen suppression, niche stabilization, and immune signaling, organic acids contribute to colonization resistance and microbial ecosystem resilience, reinforcing their role as functional postbiotic metabolites.

#### Polyamines

Polyamines, such as putrescine, spermidine, and spermine, are low-molecular-weight polycations generated through microbial metabolism in the gut. These molecules interact with nucleic acids, proteins, and cellular membranes and act essential regulators of cell proliferation, differentiation, and apoptosis in host tissues^47^. Gut microbiota-derived polyamines contribute both to maintaining luminal polyamine availability and to modulating gene expression in intestinal epithelial cells, thereby supporting barrier integrity and mucosal homeostasis. Experimental evidence highlights their metabolic relevance; for example, supplementation with L. reuteri has been shown to alleviate metabolic syndrome in murine models by increasing microbiota-derived spermidine levels, whereas inhibition of polyamine biosynthesis abolishes these beneficial effects^8^. More broadly, elevated polyamine availability has been associated with improved nutrient absorption, immune balance, and preservation of gut structure, while age-related declines in polyamine levels suggest a potential role in healthy ageing^48^.

#### Vitamins

The gut microbiota represents a significant endogenous source of several essential vitamins, particularly water-soluble B-group vitamins and vitamin K. As humans lack the biosynthetic pathways for most vitamins, microbial production constitutes an important complementary source. Numerous commensal bacteria, including LAB and bifidobacteria, possess genetic pathways enabling de novo vitamin synthesis^49^. For instance, multiple Bifidobacterium species synthesize folate (vitamin B9), with B. adolescentis and B. pseudocatenulatum identified as high-yield producers both in vitro and in vivo^44^. Similarly, Propionibacterium freudenreichii and selected Lactobacillus strains harbor genes involved in cobalamin (vitamin B12) biosynthesis, while microbial production of riboflavin (B2), pyridoxine (B6), thiamine (B1), niacin (B3), and menaquinone (vitamin K2) has also been extensively documented^49^. These microbially derived vitamins function as indispensable cofactors in host metabolism, supporting processes such as DNA synthesis and repair, redox homeostasis, energy metabolism, and blood coagulation, thereby reinforcing the nutritional and functional significance of postbiotic vitamin production.

### Cell wall components: peptidoglycans, muramyl peptides, and teichoic acids

Bacterial cell wall components represent a major class of structural postbiotics with potent immunometabolic effects. Peptidoglycan (PG) fragments derived from probiotic bacteria act as microbe-associated molecular patterns (MAMPs) capable of modulating host immunity and metabolism without microbial viability. Purified PG from Lactobacillus strains has demonstrated anti-inflammatory and metabolic benefits, including attenuation of interleukin-12 (IL-12) production and improvement of insulin sensitivity in experimental models. The minimal bioactive motif muramyl dipeptide (MDP), recognized by the intracellular receptor nucleotide-binding oligomerization domain-containing protein 2 (NOD2), plays a central role in these effects. Administration of MDP improves glucose tolerance and reduces adipose inflammation independently of microbiota compositional changes, an effect mediated through NOD2–interferon regulatory factor 4 (IRF4) signaling pathways that counteract obesity-induced insulin resistance^44,50^. These findings highlight muramyl peptides as safe and targeted postbiotic immunomodulators. TAs, including LTAs, are anionic polymers characteristic of Gram-positive bacteria that also exert context-dependent immunological effects. LTAs derived from probiotic Lactobacillus strains interact with Toll-like receptor 2 (TLR2), leading to reinforcement of epithelial barrier integrity and controlled cytokine modulation. In colitis models, probiotic LTAs upregulated tight junction proteins such as zonula occludens-1 (ZO-1) and suppressed nuclear factor kappa B (NF-κB)–driven inflammation via AMP-activated protein kinase (AMPK) and p38 mitogen-activated protein kinase (p38 MAPK) signaling pathways^51^. In contrast, structurally distinct LTAs from pathogenic bacteria elicit excessive inflammatory responses and barrier disruption. Thus, subtle structural variations in TAs determine whether immune signaling promotes tolerance or pathology, underscoring the importance of source-specific purification strategies for postbiotic applications.

### Exopolysaccharides (EPS): structure–function relationships and bioactivity

Exopolysaccharides (EPS) are high-molecular-weight polymers secreted by bacteria into the extracellular milieu and represent one of the most structurally diverse classes of postbiotics. EPS produced by LAB may be classified as homopolysaccharides or heteropolysaccharides and often contain chemical substituents such as acetyl, phosphate, or pyruvate groups. These fine structural features critically determine EPS physicochemical properties and biological activities, giving rise to well-defined structure–function relationships^52^. Modulation of fermentation conditions or EPS biosynthetic genes enables targeted production of polymers with tailored functional traits. Functionally, microbial EPS exhibit broad bioactivity, including immunomodulatory, antioxidant, antimicrobial, and microbiota-modulating effects. LAB-derived EPS interact with host immune cells, modulating cytokine responses and enhancing mucosal and systemic immunity, while others exert antioxidant effects through inhibition of NF-κB and MAPK signaling pathways. Certain EPS also interfere with pathogen adhesion and biofilm formation or act as fermentable substrates for beneficial commensals, indirectly reshaping gut microbial communities. In vivo studies further demonstrate that EPS supplementation can alleviate inflammation-driven tissue injury and improve gut resilience^44^. Collectively, the structural versatility and multifunctionality of EPS position them as particularly promising postbiotics for applications in food, nutraceutical, and pharmaceutical systems.

### Proteins and peptides: bacteriocins, bioactive peptides

**Fig. 5:**
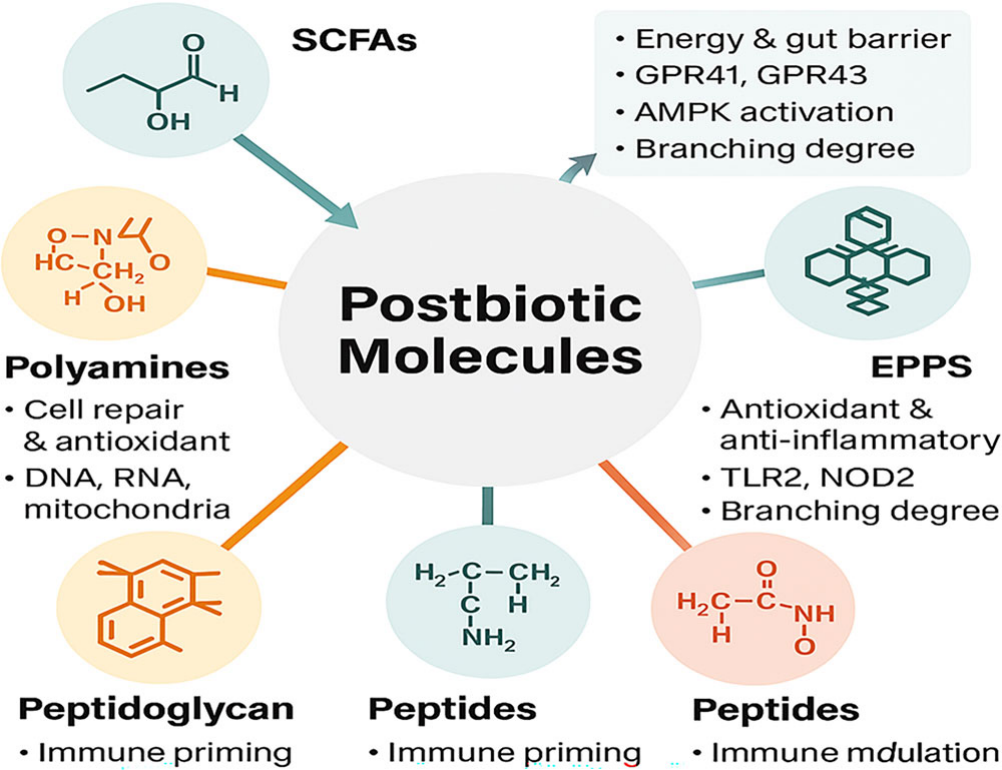
Structure–function relationship of postbiotic molecules. The diagram illustrates key classes of postbiotic compounds (short-chain fatty acids (SCFAs), polyamines, peptidoglycan, peptides, and exopolysaccharides (EPPS) radiating from a central hub. Each peripheral section displays a representative chemical or conceptual structure alongside its primary physiological roles (immune priming, cell repair, gut barrier integrity) and specific mechanistic targets (GPR41/43, AMPK, TLR2, and NOD2).

Bacteriocins are ribosomally synthesized antimicrobial peptides produced predominantly by LAB that exert narrow-spectrum bactericidal activity, primarily against closely related species. Their mechanisms include membrane pore formation or inhibition of cell wall synthesis, often through interaction with lipid II, a key precursor in bacterial cell wall biosynthesis. Well-characterized examples such as nisin produced by *Lactococcus lactis* and pediocin produced by *Pediococcus* species are approved as natural food preservatives, reflecting their stability and safety^53^. Beyond food preservation, bacteriocins are increasingly investigated as therapeutic postbiotics. They can reshape gut microbial communities by selectively suppressing pathobionts while favoring beneficial taxa, an effect associated with improved epithelial integrity and host performance in animal models. Moreover, bacteriocins have demonstrated synergistic activity when combined with conventional antibiotics, enhancing efficacy against multidrug-resistant pathogens and reducing the required antibiotic doses^54^. Importantly, bacteriocins also display immunomodulatory properties. Both in vitro and in vivo studies indicate that bacteriocin-producing *L. plantarum* strains attenuate endotoxin-induced inflammatory responses by reducing pro-inflammatory cytokine and nitric oxide production. Additional evidence shows that bacteriocins from *L. lactis* can stimulate T- and B-cell proliferation, suggesting adjuvant-like effects on adaptive immune responses. Collectively, these findings position bacteriocins not only as antimicrobial agents but also as multifunctional postbiotic peptides capable of modulating both microbiota composition and host immune responses. Figure 5 summarizes the structural basis of postbiotic activity, illustrating how chemical diversity among peptides, SCFAs, EPS, and peptidoglycan-derived fragments determines host signaling pathways and physiological outcomes.

Beyond bacteriocins, probiotic bacteria secrete a diverse repertoire of bioactive proteins and peptides that directly interact with host tissues. A prominent example is the p40 and p75 proteins secreted by *L. rhamnosus* GG, which arise from cell wall hydrolase activity. These proteins exert trophic and anti-apoptotic effects on intestinal epithelial cells by activating epidermal growth factor receptor (EGFR)–dependent signaling pathways, thereby enhancing epithelial survival, reinforcing tight junctions, and preserving barrier integrity^20,55^. In animal models, administration of purified p40/p75 proteins or the producing strain protects against chemically induced colitis by limiting epithelial cell death and enhancing mucus production. Additional proteinaceous postbiotics include surface-layer proteins (SLPs) and secreted factors that upregulate mucin expression and protect against inflammatory barrier disruption. Furthermore, microbial fermentation of dietary proteins releases small bioactive peptides with systemic effects, such as angiotensin-converting enzyme (ACE)–inhibitory or opioid-like activities, which can influence blood pressure regulation and host physiology. Together, these protein and peptide- derived postbiotics represent a versatile class of microbial effectors that deliver barrier-protective, immunomodulatory, and metabolic benefits without the need for live microbial administration^56^.

### Stress–response and signaling effectors

Beyond cell-wall fragments and metabolites, intracellular stress-response proteins released after probiotic inactivation (thermal treatment) represent an additional, mechanistically plausible class of immunoactive postbiotic candidates. Among these, bacterial heat-shock proteins (HSPs) such as GroEL, a member of the Hsp60 family, are canonical molecular chaperones that support protein folding and stress tolerance in bacteria, yet they may also contribute to host immune sensing when exposed to mucosal immune cells. Importantly, the immunomodulatory activity attributed to heat-killed probiotics should not be automatically assigned to HSPs alone, because inactivation simultaneously increases the accessibility of multiple MAMPs, including peptidoglycan fragments, LTAs, and bacterial nucleic acids, which can act synergistically with stress proteins. Nevertheless, several studies suggest that heat-inactivated *Lactobacillus* preparations can retain specific innate immune outputs compared with their live counterparts, including increased macrophage and dendritic cell responsiveness and higher cytokine production under defined stimulation conditions^57,58^. For instance, heat-treated *L. casei* Zhang has been reported to enhance immune activation markers and cytokine profiles relative to the viable form, supporting the concept that heat-induced exposure of intracellular immunogenic components may increase host recognition^59^.

In parallel, mixtures of heat-killed LAB and yeast have been shown to stimulate cytokine responses such as interleukin-1β (IL-1β) and interleukin-23 (IL-23) in peripheral immune cell models, consistent with the engagement of inflammatory sensing pathways that may involve, among other effectors, stress-related proteins and downstream inflammasome-associated signaling^60^. From a translational perspective, HSP-containing paraprobiotic preparations may provide a route to immune priming without microbial replication, which could be advantageous in populations where live microorganisms raise safety or stability concerns. However, because HSPs are highly conserved proteins, immune outcomes may vary from inflammatory activation to tolerance-like responses, depending on the host context, dose, mucosal barrier status, and the accompanying MAMP background^61^. Overall, bacterial HSPs should be viewed as context-dependent contributors to the bioactivity of inactivated probiotics rather than standalone determinants, and future work should prioritize fractionation-based validation strategies to disentangle HSP-specific effects from co-exposed cell components.

### Stability, bioavailability, and structure–function determinants

A major technological advantage of postbiotics over live probiotics is their greater formulation robustness, since bioactivity is not dependent on microbial viability. As a result, many postbiotic preparations can better tolerate industrial constraints such as thermal treatment, dehydration, pressure, and extended storage, enabling improved batch-to-batch standardization and more consistent dosing compared with viability-limited probiotic products^62,63^. However, stability is highly molecule dependent, while EPS and certain cell-derived structures can remain resilient, labile peptides, enzymes, and redox-sensitive metabolites may still undergo degradation unless protected during processing and storage^63^. Following oral intake, the effective bioavailability of postbiotics is primarily governed by their site of action, because many postbiotic effects are exerted locally within the gastrointestinal tract through modulation of epithelial barriers, mucosal immune signaling, or microbial ecology rather than requiring systemic uptake. In contrast, small soluble compounds can be absorbed and influence host physiology beyond the gut, notably through portal circulation and liver-associated metabolic pathways^64^. To maximize delivery to relevant intestinal compartments, formulation strategies such as enteric coatings, biopolymer carriers, and encapsulation systems are increasingly used to control release kinetics and protect pH- or enzyme-sensitive bioactive compounds^64^. In this context, certain EPS may serve a dual role as both bioactive molecules and protective matrices, forming hydrogels or nanoscale assemblies that improve stability and support targeted colonic delivery, as shown for heteropolysaccharide EPS produced by *Weissella* spp^65^.

At the mechanistic level, postbiotic efficacy is strongly shaped by structure–function relationships, where subtle chemical variations can produce disproportionate changes in bioactivity. For EPS, parameters such as monosaccharide composition, molecular weight distribution, branching degree, and functional substitutions (acetylation or phosphorylation) modulate immunomodulatory potential, physicochemical behavior, and antioxidant properties. In particular, uronic acid–containing polymers are frequently associated with enhanced metal-chelating and radical-scavenging capacity under defined assay conditions^66^. Similarly, in peptides and proteins, even minor sequence changes or conformational shifts may alter receptor engagement and downstream signaling strength, whereas structural modifications of cell-wall-associated molecules can markedly influence innate immune recognition and pattern-recognition signaling outcomes^66^. Collectively, these principles support a rational development pipeline in which fermentation conditions, controlled processing, and targeted formulation are optimized to generate stable, mechanistically defined, and reproducible postbiotic interventions for food and therapeutic applications^66^.

## Mechanisms of action of postbiotics

Postbiotics exert biological effects through defined host sensing systems and intracellular signaling networks, rather than through microbial colonization, replication, or ecological persistence. Accordingly, the mechanistic framework of postbiotic action is best interpreted as signal–receptor–pathway coupling, where microbial-derived effectors interact with epithelial, immune, metabolic, and neuroimmune targets to shape barrier integrity, inflammatory tone, and systemic physiology. To avoid redundancy with the “Biochemistry of Postbiotic Molecules” section, the present section focuses strictly on host pathways, molecular logic, and functional outcomes, emphasizing how postbiotic stimuli converge on conserved regulatory hubs that govern inflammation, metabolism, oxidative stress, and inter-organ communication **(**Table 3 and Table 4**)**.

**Table 3:** Molecular mechanisms through which postbiotic molecules influence host physiology

Mechanistic Category	Representative Postbiotic Components	Host Cellular/Molecular Targets	Principal Signaling Pathways	Biological Effects and Functional Outcomes	References
Gut Barrier Reinforcement	SCFAs (butyrate, acetate), EPS, peptides (p40/p75), polyamines	Tight junction proteins (ZO-1, occludin, claudin-1); mucins (MUC2); AMPK	AMPK, PPARγ, MAPK, MLCK suppression	Strengthens epithelial barrier integrity, enhances mucin secretion, reduces permeability (“leaky gut”)	^44,67,218^
Immune Modulation	Peptidoglycan (MDP), LTAs, cell-wall fragments	PRRs (TLR2, NOD2, Dectin-1) on dendritic and epithelial cells	NF-κB, MAPK, STAT3, Nrf2	Balances pro/anti-inflammatory cytokine release ( ↓ TNF-α, IL-6; ↑IL-10, TGF-β); induces immune tolerance	^82^
Antioxidant and Anti-inflammatory Activity	EPS, polyphenolic metabolites, antioxidant enzymes (SOD, CAT), SCFAs	ROS/RNS species, Nrf2/Keap1 complex, mitochondrial redox systems	Nrf2–ARE, NF-κB inhibition, MAPK	Suppresses oxidative stress, upregulates antioxidant enzymes, attenuates lipid peroxidation and inflammation	^69,71,148^
Metabolic Regulation	SCFAs, peptides, polyamines, B-vitamins	Enteroendocrine cells (L-cells), adipocytes, hepatocytes	AMPK, GPR41/43, GLP-1, PPARα/γ, FGF21	Improves glucose homeostasis, lipid metabolism, energy expenditure; enhances insulin sensitivity	^44,219^
Antimicrobial Action	Bacteriocins (nisin, pediocin), organic acids, quorum-sensing inhibitors	Pathogenic cell membranes, quorum-sensing receptors	Membrane pore formation, pH disruption, AI-2 signaling interference	Inhibits pathogen growth, biofilm formation, and virulence factor expression	^2,11^
Neuroimmune and Gut–Brain Axis Effects	SCFAs, indoles, polyamines, microbial peptides	Enteric nervous system, vagus nerve, microglia	GABAergic, serotonergic, HPA-axis modulation, BDNF signaling	Improves mood and cognition, reduces neuroinflammation, supports stress resilience	^69,72^
Epigenetic and Transcriptomic Modulation	SCFAs (butyrate), polyamines, quorum-sensing peptides	HDACs, miRNA expression, chromatin structure	HDAC inhibition, DNA methylation regulation	Modulates host gene expression, enhances antioxidant and anti-inflammatory gene networks	^44,220^
Microbiota-Mediated Cross-Talk	SCFAs, bacteriocins, EPS	Gut microbial consortia, competitive microbial niches	Cross-feeding, quorum sensing, metabolite exchange	Promotes beneficial microbiota composition, suppresses pathobionts	^67,221^

**Table 4:** Postbiotic mechanisms, targets, effects, and applications

Postbiotic class	Main receptors/targets	Key signaling pathways	Major host effects	Translational applications	References
SCFAs (butyrate, acetate, propionate)	FFAR2/3 (GPCRs), AMPK, epithelial cells, immune cells	AMPK↑ (FA oxidation, ↓ lipogenesis); HDAC inhibition; GLP-1/PYY secretion; ↑ TJ proteins (ZO-1, occludin, claudins)	Reinforced gut barrier; ↓ systemic inflammation; ↑ insulin sensitivity; gut–brain axis modulation	IBD, obesity/Type 2 diabetes mellitus (T2DM), NAFLD/MASLD, dysbiosis	^70,73,75,222,223^
Exopolysaccharides (EPS) (homo-/heteropolymers)	TLR2/4, Nrf2/HO-1, NF-κB, MAPK; goblet cells (MUC2)	Nrf2/HO-1↑ (antioxidant); NF-κB/MAPK↓ (anti-inflammatory); ↑ mucin (MUC2); ↑ TEER	↓ IL-6/TNF-α/IL-1β; ↓ ROS; ↑ mucus barrier	IBD, cardiovascular protection, neuro-glial protection	^215,224^
Peptidoglycan/MDP fragments	NOD2 (RIP2), cross-talk with TLRs	IL-10/Treg induction; autophagy↑ (LC3 ↑ , p62 ↓ ); counter-regulation of TLR-IFN via IRF4/DUBA	Immune tolerance; mucosal healing; ↓ permeability	IBD; metabolic endotoxemia; immune balance	^225–227^
Bacteriocins & CFS/EVs (nisin, lactocins; supernatants; extracellular vesicles)	Pathogen membranes; immune PRRs; Nrf2 in microglia	Direct antimicrobial + immunomodulation; Nrf2-SOD1↑ in microglia; ↓ NO	↓ pathogen burden; neuro-/immune modulation without colonization	Mucosal infections; IBD; neuro-inflammation	^60,228,229^
Polyamines (spermidine, spermine, putrescine)	Autophagy (ATG), Nrf2/HO-1, mitochondria	↑ Autophagy; ↑ Nrf2-GPX4; ↓ oxidative stress; ↑ synaptic plasticity	Neuroprotection; epithelial barrier support; energy metabolism	Healthy aging; neurodegeneration; mucosal integrity	^47,230–232^
Whole-cell & multi-strain postbiotics (pasteurized Akkermansia)	Amuc_1100/OMPs, EPS, SCFAs; PRRs & GPCRs	Synergy barrier–immune–metabolism; ↓ LPS; ↓ adipose inflammation	↑ Insulin sensitivity; ↓ steatosis; ↓ pro-inflammatory cytokines	Obesity, T2DM, NAFLD, IBD	^56,60,233^
Multi-molecule “cocktails” (SCFAs + EPS + MDP + peptides)	Convergence FFAR2/3–AMPK–Nrf2–NF-κB–NOD2	Additive/synergistic effects: ↑ barrier, ↑ immune tolerance, ↑ antioxidant defense, ↑ metabolic balance	Multi-target correction of complex diseases	Metabolic syndrome, IBD, cardiovascular disease (CVD), neuro-inflammation	^76^

### Gut barrier integrity

A central functional consequence of postbiotic exposure is the strengthening of intestinal barrier integrity, which depends on coordinated regulation of tight junction architecture, cytoskeletal contractility, and mucus-layer homeostasis. Mechanistically, postbiotics improve barrier function by activating intracellular pathways that stabilize junctional complexes and suppress permeability-promoting signals. One recurrent mechanism involves AMPK activation, which reduces junction destabilization by limiting myosin light chain kinase (MLCK)-driven actomyosin contraction, thereby preserving tight junction localization and continuity^67,68^. In parallel, phosphoinositide 3-kinase/protein kinase B (PI3K/Akt) signaling supports membrane retention and phosphorylation-dependent stabilization of junctional proteins, while epigenetic modulation can enhance transcriptional accessibility of epithelial barrier genes under inflammatory pressure^69,70^.

Barrier reinforcement is also achieved through regulation of the mucus layer, which is essential for maintaining spatial segregation between luminal microbes and the epithelial surface. Postbiotic stimuli can promote goblet cell differentiation and activation of mucin gene expression programs through extracellular signal–regulated kinase 1/2 (ERK1/2) signaling and controlled engagement of inflammatory transcriptional circuits, resulting in increased mucus thickness and improved resistance to microbial adhesion or invasion^44,71^. Functionally, these changes are captured by reproducible readouts, including increased transepithelial electrical resistance (TEER), reduced paracellular permeability, and lower systemic exposure to microbial inflammatory triggers^67^. Across inflammatory and metabolic disease models, barrier reinforcement interrupts self-amplifying loops linking permeability to endotoxemia and mucosal inflammation. In intestinal inflammation, restoration of epithelial cohesion reduces antigen penetration and inflammatory escalation, while in metabolic disorders, stabilization of gut permeability limits systemic inflammatory spillover that contributes to insulin resistance and tissue dysfunction^72,73^.

### Immune modulation

**Fig. 6:**
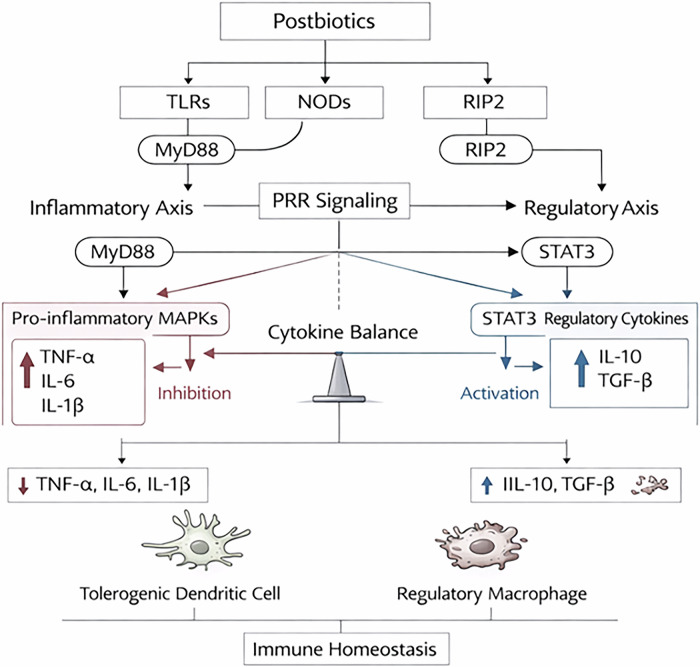
Postbiotic-driven immune modulation via biased TLR2 and NOD2 signaling pathways. The flowchart delineates the engagement of pattern recognition receptors (TLRs, NODs, RIP2) by postbiotics, which splits into inflammatory (MyD88) and regulatory (STAT3) signaling axes. This PRR signaling shifts the overall cytokine balance, leading to the inhibition of pro-inflammatory markers and the activation of regulatory cytokines. Upward (↑) and downward (↓) arrows denote the respective increase (IL-10, TGF-β) or decrease (TNF-α, IL-6, IL-1β) in cytokine expression, culminating in the promotion of tolerogenic dendritic cells, regulatory macrophages, and overall immune homeostasis.

Postbiotic-driven immunomodulation is primarily mediated through pattern-recognition receptor (PRR) signaling, where innate sensors interpret microbial cues and generate calibrated downstream responses. Unlike invasive pathogens that often trigger robust inflammatory activation, postbiotics more frequently induce biased PRR signaling, characterized by restrained inflammatory intensity and enhanced resolution programming **(**Fig. 6**)**. This is reflected in controlled modulation of NF-κB transcriptional amplitude and a shift toward regulatory cytokine dominance, supporting immune homeostasis rather than immunopathology^69,74^. At the intracellular signaling level, PRR engagement regulates a balance between inflammatory and regulatory programs through coordinated control of NF-κB, mitogen-activated protein kinases (MAPKs) (p38/JNK/ERK), signal transducer and activator of transcription 3 (STAT3), and cytoprotective transcriptional pathways^71,75^.

A recurring mechanistic pattern is suppression of NF-κB nuclear translocation and attenuation of MAPK signaling amplification, which reduces transcription of pro-inflammatory mediators, while STAT3-dependent stabilization of regulatory immune phenotypes contributes to long-term immune tolerance^67,69^. These effects translate into an immunological signature typically characterized by increased anti-inflammatory cytokines (interleukin-10 (IL-10) and TGF-β) and reduced pro-inflammatory mediators (tumor necrosis factor-α (TNF-α), IL-6, and IL-1β) across diverse experimental contexts^73,76^. Importantly, postbiotics also reshape immune cell programming. Dendritic cells exposed to postbiotic cues can adopt tolerogenic phenotypes, characterized by reduced IL-12, and increased IL-10 production, thereby favoring expansion of regulatory T-cell responses over inflammatory effector polarization. Likewise, macrophage responses may shift toward enhanced pathogen-handling competence with limited collateral inflammatory damage, providing a mechanistic basis for immune homeostasis in chronic inflammatory conditions^44^. These properties support broad relevance in inflammation-driven disorders, including colitis, metabolic inflammation, autoimmune diseases, and allergic immune dysregulation^72,77^.

### Metabolic regulation

Postbiotics influence metabolic homeostasis by targeting conserved regulatory hubs that coordinate nutrient sensing, lipid flux, glucose utilization, and endocrine feedback. At the core of this network is AMPK, a master energy sensor that integrates nutrient availability with anabolic–catabolic balance. Postbiotic-driven AMPK activation promotes fatty acid oxidation and reduces de novo lipogenesis through inhibition of lipogenic transcriptional regulators, while also enhancing insulin sensitivity via improved glucose uptake and greater metabolic flexibility^67,78^. In parallel, nuclear receptor–mediated pathways support mitochondrial biogenesis, oxidative metabolism, and reduced ectopic lipid accumulation, contributing to protection against hepatic steatosis and adipose dysfunction^44,75,79^.

A second major component of postbiotic metabolic action involves gut–endocrine signaling. By stimulating enteroendocrine pathways, postbiotics enhance secretion of gut hormones such as glucagon-like peptide-1 (GLP-1) and peptide YY (PYY), improving glycemic control, satiety, and energy regulation through systemic hormonal and neural circuits^80^. Functionally, these mechanisms collectively map onto consistent metabolic endpoints, including improved insulin sensitivity, reduced systemic inflammatory tone, improved lipid profiles, and reduced hepatic lipid accumulation in experimental models of metabolic dysfunction^72^.

### Antioxidant and anti-inflammatory pathways

**Fig. 7:**
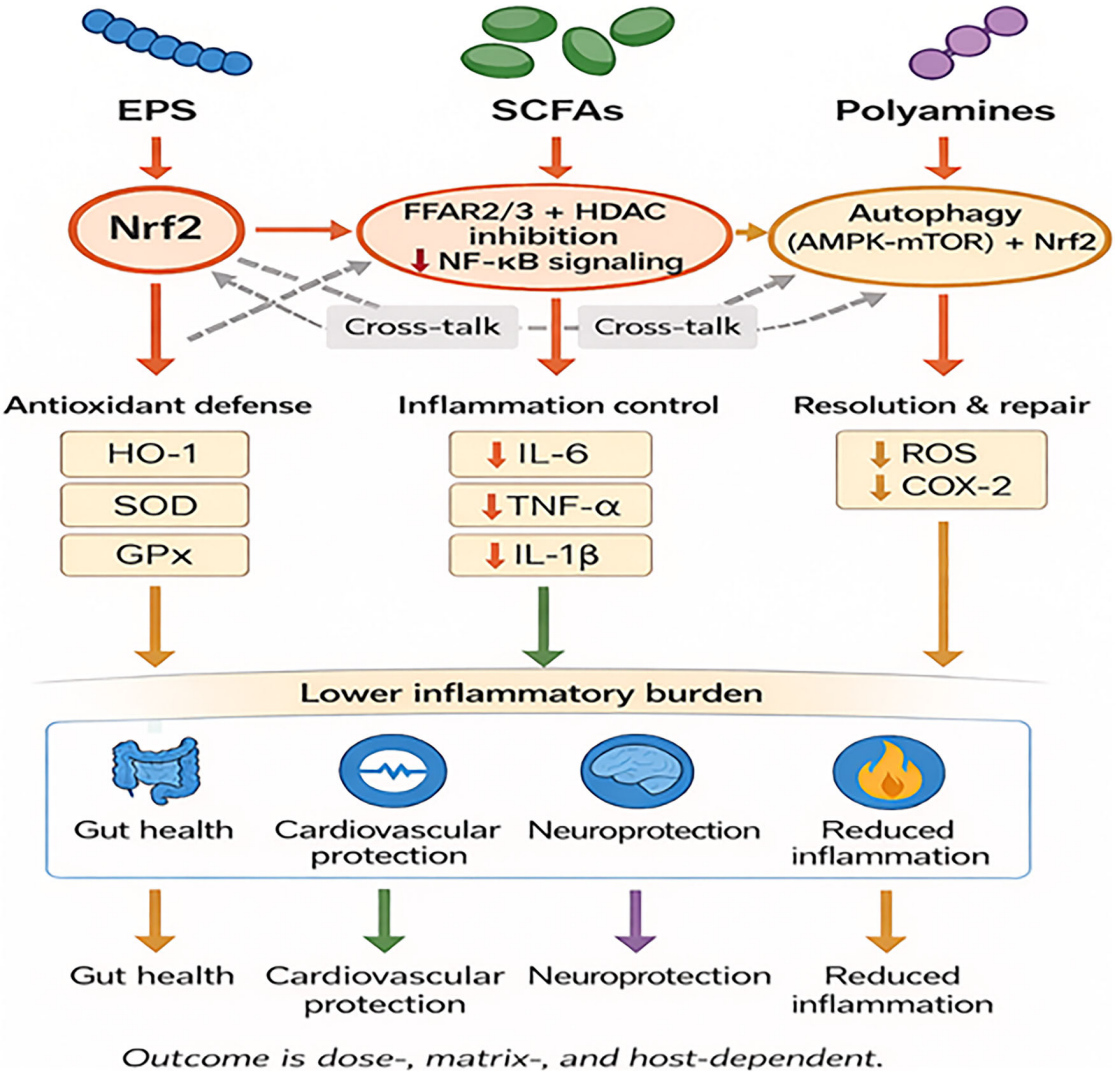
Antioxidant and anti-inflammatory pathways. The schematic illustrates how specific postbiotic components (EPS, SCFAs, and polyamines) trigger distinct intracellular mechanisms, including Nrf2 activation, FFAR2/3 engagement with HDAC inhibition, and autophagy. These signaling pathways promote antioxidant defense, control inflammation, and facilitate tissue resolution and repair, ultimately culminating in a lower systemic inflammatory burden that supports gut, cardiovascular, and neurological health. Downward arrows (↓) denote the reduction or inhibition of specific inflammatory cytokines and reactive markers, while dashed gray arrows represent mechanistic cross-talk between the primary signaling pathways.

Oxidative stress and inflammation form a tightly linked pathophysiological axis in chronic disease progression. Postbiotics modulate this axis through dual regulation of cytoprotective antioxidant defenses and inflammatory transcriptional amplification (Fig. 7). A principal mechanism is activation of the nuclear factor erythroid 2–related factor 2–antioxidant response element (Nrf2–ARE) pathway, which induces antioxidant genes and enhances resilience to oxidative injury by restoring redox balance and limiting mitochondrial dysfunction^44,67^. At the same time, postbiotics frequently suppress inflammatory escalation through inhibition of NF-κB and MAPK signaling, reducing cytokine burden and preventing tissue damage caused by prolonged inflammatory activation^76,81^. These mechanisms have consistent outcomes across pathological contexts, including accelerated mucosal repair during intestinal inflammation, attenuation of systemic oxidative stress in metabolic dysfunction, and reduced inflammatory injury across vascular and neuroimmune systems^50,72,73,82^.

### Gut–brain axis and neuroimmune signaling

Postbiotic effects extend beyond the intestine through integration into gut–brain communication pathways that connect microbial sensing to neuroimmune, endocrine, and neural responses. Mechanistically, postbiotics shape this axis through combined regulation of systemic inflammatory tone, endocrine mediators, and neuroimmune signaling processes. One key feature is reduction of peripheral pro-inflammatory cytokine spillover, which lowers neuroinflammatory priming and limits microglial activation in chronic inflammatory states^73,76^. Postbiotics also influence neurophysiology through epigenetic mechanisms, notably histone deacetylase (HDAC)-linked regulation in certain contexts, affecting expression of neurotrophic and stress-response pathways such as brain-derived neurotrophic factor (BDNF) programs that support plasticity and resilience^83^. At the neuroendocrine level, enteroendocrine coupling contributes to appetite, mood, and stress regulation through hormone release and vagal-afferent signaling pathways. These combined mechanisms provide a framework for interpreting evidence linking postbiotic exposure with improved cognitive performance, reduced anxiety-like behavior, and neuroprotective outcomes in experimental models, while emphasizing the need for scalable human validation^44,82^.

### Cross-talk between molecules: synergistic actions

**Fig. 8:**
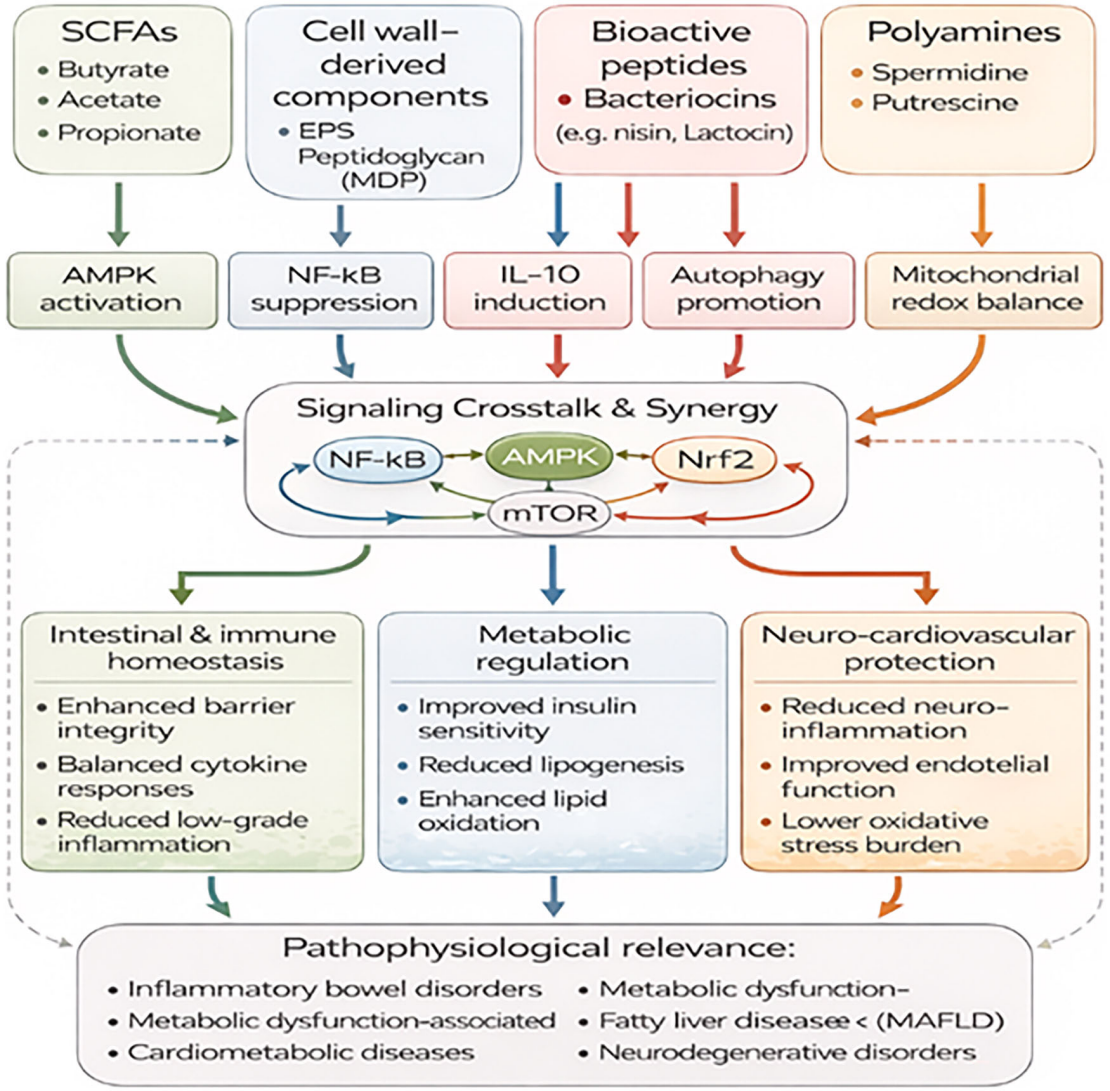
Synergistic actions of postbiotics. The flowchart shows how various postbiotic molecules trigger cellular responses that converge into a central signaling network (NF-kB, AMPK, Nrf2, mTOR). This integrated crosstalk drives intestinal, metabolic, and neuro-cardiovascular homeostasis to help manage various pathophysiological disorders. Solid arrows indicate direct signaling pathways and physiological effects, while dashed lines represent systemic connections.

A defining feature of postbiotic activity is that outcomes rarely arise from a single isolated signaling pathway. Instead, postbiotics function as systems-level modulators, in which multiple molecular inputs converge on shared regulatory nodes controlling barrier integrity, inflammatory calibration, metabolism, and redox stability. Mechanistic synergy emerges through coordinated engagement of interconnected pathways^71,72^. This convergence provides a mechanistic rationale for observations that complex postbiotic preparations can outperform purified individual constituents in multifactorial disease models, because multi-target pathway engagement corrects pathological networks rather than a single endpoint^56^ (Fig. 8).

## Paraprobiotics: inanimate cells with biochemical activity

### Definition and scope (inactivated but intact microbes)

In this review, paraprobiotics are discussed as cell-based, non-viable microbial preparations that retain biologically relevant structural features and therefore occupy an intermediate space between live probiotics and cell-free postbiotic fractions. Their functional relevance stems primarily from the preserved presentation of cell-envelope-associated constituents, including peptidoglycan-associated motifs, TAs, SLPs, and membrane-associated polysaccharides, which can remain accessible to host sensing systems even in the absence of replication or active metabolism^25,84^. This concept is aligned with the *“*probiotic paradox*”*, whereby probiotic-associated effects may persist following loss of viability^5,25^. From a translational and manufacturing perspective, paraprobiotics provide a technology-compatible strategy for food and nutraceutical systems in which microbial survival is difficult to ensure, including products exposed to thermal, pressure, dehydration, or mechanical stresses, or settings in which safety constraints limit the administration of live microorganisms^84^. Importantly, paraprobiotic preparations are not uniform biological entities. Their biological performance depends on the inactivation approach, the physiological state of cells at the time of treatment, and strain-specific cell wall organization, which collectively determine whether cells remain morphologically intact, become partially permeabilized, or fragment into envelope-derived particulate assemblies. This structural outcome governs the exposure and spatial organization of MAMPs, thereby shaping downstream host recognition pathways despite the absence of microbial viability^25^.

### Inactivation methods (heat, irradiation, high pressure, sonication)

Paraprobiotics are produced through controlled inactivation of microbial cells using physical or physicochemical treatments that abolish viability while preserving, to varying degrees, structurally relevant cellular attributes. Major industrial and experimental approaches include thermal processing, ionizing irradiation, high-pressure processing (HPP), and sonication, each inducing distinct cellular damage patterns and generating method-specific structural signatures in paraprobiotic preparations^84^. Importantly, these treatments do not simply “kill” microorganisms; rather, they condition the resulting biological behavior by modulating cell-envelope integrity, permeability, and molecular accessibility (Table 5). Thermal inactivation remains the most widely implemented strategy due to its scalability and reproducibility. Elevated temperatures induce irreversible protein unfolding, membrane destabilization, and nucleic acid damage, leading to rapid loss of microbial viability^48^. Moderate conditions such as pasteurization-like treatments may preserve substantial portions of cell wall structure and surface macromolecules, whereas more severe heating increases sterility assurance at the cost of heat-labile cellular constituents, including enzymes and certain surface-associated proteins or polysaccharides. Thus, heat treatment requires careful optimization to balance effective inactivation with retention of structurally mediated bioactivity. Ionizing irradiation (commonly γ-rays) primarily inactivates cells through DNA strand breaks and oxidative base damage, offering a non-thermal mechanism for microbial replication arrest^47^.

**Table 5:** Inactivation methods used to produce paraprobiotics: mechanisms, advantages, limitations, and recent applications

Method	Mechanism of inactivation	Advantages	Limitations	References
Heat treatment (pasteurization, autoclaving)	Denaturation of cytosolic and membrane proteins; disruption of lipid bilayers; fragmentation of DNA and RNA; irreversible loss of enzymatic activity	Simple and reproducible; easily scalable; ensures sterility; compatible with most food matrices; high dose standardization	Degradation of heat-labile bioactives (enzymes, peptides); alteration of metabolomic profile; reduced antioxidant capacity	^17,84,126,234^
Irradiation (γ-rays, electron beams)	Single- and double-strand DNA breaks; intracellular oxidative stress; loss of replication capacity without extensive cell lysis	Non-thermal; deep penetration; preservation of surface structures (proteins, LTAs); retained immunomodulatory potential	High cost and regulatory constraints; possible degradation of EPS and peptidoglycan at high doses; variable consumer acceptance	^85,235^
High-pressure processing (HPP)	Membrane permeabilization; denaturation of membrane-associated proteins; disruption of enzymatic complexes; metabolic arrest	Non-thermal; excellent preservation of heat-sensitive molecules; industrially implemented; retention of surface antigens	High capital investment; narrow pressure–time–temperature window; partial cell lysis at extreme pressures	^236–239^
Sonication (ultrasound > 20 kHz)	Acoustic cavitation; shear forces; controlled membrane rupture; release of cell wall fragments (peptidoglycan, TAs)	Generation of targeted bioactive fragments; enhanced exposure of MAMPs; strong therapeutic and immunomodulatory potential	Not always fully sterilizing; possible generation of free radicals; requires precise control of parameters	^240,241^
Mild chemical treatment (ethanol, organic acids)	Membrane disorganization; partial protein precipitation; inhibition of metabolic pathways	Simple and low-cost; non-thermal; suitable for small-scale or laboratory applications	Potential chemical residues; alteration of surface structures; regulatory limitations	^17,84,240^
UV / pulsed light	Formation of pyrimidine dimers; oxidative DNA damage; inhibition of DNA replication	Rapid processing; low energy consumption; non-thermal	Limited penetration depth; reduced efficacy in opaque matrices; risk of incomplete inactivation	^242–246^

At controlled doses, irradiation can maintain cellular morphology and membrane architecture, enabling homogeneous inactivation even within complex matrices. However, excessive dosing may generate reactive chemical species that alter envelope polymers such as peptidoglycan and EPS, and membrane lipids, with potential implications for functional performance and sensory stability in food matrices^85^. HPP typically operates at 400–600 MPa and inactivates cells through membrane permeabilization, protein conformational disruption, and collapse of intracellular homeostasis, often under low-to-moderate temperature conditions^47^. This non-thermal profile may favor retention of thermolabile surface components relative to heat-based inactivation. Nonetheless, prolonged exposure or excessive pressure may induce partial lysis or irreversible deformation of envelope structures, emphasizing the need for strain-specific and application-guided process optimization. Sonication relies on acoustic cavitation, producing localized shear forces and transient pressure gradients capable of perforating or fragmenting microbial cells^48^. Unlike heat or HPP, sonication frequently generates heterogeneous outcomes, ranging from permeabilized intact cells to extensively fragmented cellular material. While it can reduce viable counts, sonication alone may not always achieve complete microbial inactivation and is therefore often combined with complementary treatments. In addition, overexposure may promote free radical formation and oxidation of sensitive components, which warrants attention during postbiotic formulation design^85^.

### Biochemical consequences of inactivation

The conversion of viable probiotics into paraprobiotics is not a strictly binary “live-to-dead” transition; rather, it produces method-dependent structural and biochemical fingerprints that dictate which microbial constituents are preserved, modified, or exposed in the final preparation. Depending on the type of inactivation and its intensity, such as heat, high pressure, and ultrasound, paraprobiotics may retain intact cell envelopes, partially fragmented structures, or released intracellular components, with consequent differences in functional profiles. These graded structural outcomes underlie the heterogeneity in observed biological responses and reinforce the importance of characterizing inactivation-specific microstructural features when defining paraprobiotic effects^85–87^. Under mild-to-moderate inactivation conditions, viability can be abolished while maintaining a largely preserved envelope architecture, such that major cell-associated structures remain spatially organized in a particulate cellular format^47^. In contrast, harsher physical treatments such as prolonged ultrasonication or excessive pressure promote partial disruption of the cell wall and membrane, yielding preparations enriched in soluble structural fragments, including peptidoglycan subunits, teichoic acid fragments, and, in Gram-negative bacteria, lipopolysaccharide-associated motifs, together with varying levels of intracellular macromolecule leakage^88^.

Beyond gross structural preservation versus fragmentation, inactivation can markedly alter molecular accessibility, particularly for surface-anchored and membrane-embedded proteins. Heat and pressure treatments may induce partial unfolding or lipid bilayer rearrangements, unmasking domains that are less accessible in live cells, whereas irradiation can detach or solubilize specific SLPs fractions while maintaining elements of higher-order structure^48^. These shifts influence not only immune sensing but also the physicochemical behavior of the preparation, including particle size distribution, aggregation propensity, and sedimentation kinetics, all of which may affect delivery and interaction with host tissues. Inactivation can introduce chemical modifications that reshape functional stability. High-temperature treatments may promote protein denaturation, loss of labile metabolites, or non-enzymatic reactions such as glycation, whereas non-thermal approaches reduce heat-driven damage but may introduce oxidative modifications through radical formation^85^. For example, moderate heating (70–90 °C) may preserve antioxidant capacity, while autoclaving can substantially alter polysaccharide structures and reduce bioactivity^47^. Similarly, irradiation at excessive doses has been associated with degradation of EPS and weakening of peptidoglycan networks, reinforcing the need for careful dose optimization^85^. Collectively, these processing- driven structural and biochemical fingerprints provide a rational framework for the intentional design of paraprobiotic preparations with reproducible composition and function, and they explain why inactivated probiotics cannot be considered interchangeable entities across different inactivation methods or microbial strains, even when derived from the same parent species^86,89^.

### Safety advantages over live probiotics

Paraprobiotics offer a distinct safety advantage over live probiotics because their non-viable nature eliminates replication-dependent risks, including uncontrolled microbial overgrowth, persistence within host tissues, and systemic dissemination. While probiotics are generally well tolerated in healthy populations, safety concerns have been reported in vulnerable patient groups. Clinical data demonstrate that probiotic-associated bloodstream infections, particularly in intensive care unit patients with central venous catheters, are associated with significantly increased mortality, highlighting clinically relevant risks linked to live probiotic administration in high-risk settings^90,91^. Inactivated preparations inherently mitigate these concerns by eliminating the possibility of microbial colonization, persistence, and bloodstream invasion. A further and increasingly important safety dimension relates to horizontal gene transfer and antimicrobial resistance (AMR). Whole-genome analyses have demonstrated that some live probiotic strains harbor transferable AMR genes, raising concerns about their potential dissemination within the gut microbiome. As a result, regulatory frameworks now emphasize strain-level genomic characterization and explicit exclusion of acquired antimicrobial resistance determinants as a prerequisite for probiotic authorization, reinforcing the safety rationale for non-viable alternatives such as paraprobiotics^3,13^.

Because paraprobiotics lack the capacity for replication, they present a substantially reduced risk of AMR dissemination, thereby simplifying risk–benefit assessment in sensitive clinical, pediatric, and food applications. From an industrial perspective, paraprobiotics also offer major technological advantages related to dose consistency and formulation stability. In contrast, live probiotic products frequently undergo viability loss during processing, distribution, and storage, resulting in variable biological activity and increased quality-control constraints. Inactivated preparations preserve a stable functional dose throughout shelf life, typically eliminate the need for cold-chain logistics, and reduce batch-to-batch variability. Their non-metabolically active nature further prevents unintended fermentation, gas formation, or matrix alterations during storage, thereby improving manufacturability, product robustness, and consumer acceptability^2,3^. Because paraprobiotics do not undergo metabolic activity or proliferation, they offer greater predictability of biological behavior, as they do not engage in growth-dependent metabolic interactions with substrates, medications, or resident microbiota. This metabolic inertness enhances reproducibility in food and clinical applications and supports their use when safety margins must be tightly controlled, particularly in vulnerable populations^85,92^.

### Applications in vulnerable populations (infants, elderly, immunocompromised)

The improved safety and formulation predictability of paraprobiotics make them particularly suitable for vulnerable populations, including infants, older adults, and immunocompromised or critically ill individuals, for whom the use of live probiotics may raise disproportionate risk despite potential benefits^25^. In these settings, a pragmatic advantage of paraprobiotics is their ability to deliver microbial-derived structural cues without introducing organisms capable of replication, persistence, or systemic translocation. In early life, immune immaturity and intestinal vulnerability increase susceptibility to gastrointestinal disorders such as necrotizing enterocolitis (NEC). While live probiotics have been explored for prevention, sporadic reports of probiotic-associated sepsis in preterm infants highlight the need for safer alternatives. Paraprobiotics may support gut homeostasis and immune maturation without infection risk, and emerging evidence suggests that inactivated microbial preparations can influence intestinal and immune parameters in infants with favorable tolerability profiles^93,94^.

These properties support their consideration in infant nutrition systems where safety assurance is paramount. Ageing is characterized by immunosenescence, chronic low-grade inflammation, and increased susceptibility to infections. In frail older adults, probiotic use is often approached cautiously due to comorbidities and the theoretical risk of bacteremia. Paraprobiotics provide a non-replicating format that may still modulate immune tone and gut function. Preclinical work indicates that heat-killed LAB can enhance innate immune markers and infection resistance in aged animal models, while early human findings suggest potential benefits for stress-related outcomes and inflammatory status^85,88^. In immunocompromised populations, live probiotics may be contraindicated due to infection risk. Paraprobiotics represent a viable alternative to support barrier function and immune balance without introducing replicating microbes. In immunosuppressed animal models, heat-killed probiotic strains have been reported to improve immune parameters and mitigate inflammatory dysregulation without evidence of infectious complications^95^.

## Food matrix effects on postbiotic stability and gastrointestinal release

The commercialization of functional biotics, such as probiotics, prebiotics, and more recently postbiotics, has been accelerated by the increasing interest in microbiome-targeted interventions. In comparison to live probiotics, postbiotics provide a number of technological benefits, such as increased stability during processing and storage, a longer shelf life, and a reduction in safety concerns related to microbial viability^81^. These characteristics permit their integration into a wide range of food matrices, including dairy products, beverages, cereals, and nutraceutical formulations. The increasing consumer awareness of gut health and preventive nutrition is driving rapid growth in the functional food market and stimulating industrial interest in microbiome-derived bioactive ingredients^36^. Consequently, postbiotic-based products are increasingly being explored as next-generation functional ingredients for applications in functional foods, dietary supplements, and clinical nutrition. However, further regulatory harmonization, standardized characterization methods, and robust clinical validation remain necessary to fully realize their commercial potential^2,96^.

### Dairy and fermented foods

Fermented dairy products are highly effective matrices for postbiotic delivery because their protein–lipid microstructure can enhance stability during processing/storage and modulate gastrointestinal release dynamics. Yogurt, kefir, and cheese naturally accumulate fermentation-derived metabolites, including organic acids, bacteriocins, EPS, and proteolysis-derived peptides, which support both technological performances including texture, and preservation, and bioactivity^97^. Rather than acting as passive carriers, dairy matrices can protect sensitive postbiotic fractions through gel-like protein networks and colloidal structures that limit premature degradation and help maintain functional properties during shelf life. During digestion, the dairy matrix structure can shape release kinetics, gastric coagulation and buffering may delay diffusion and protect labile components, whereas intestinal proteolysis progressively dismantles the matrix and promotes the release of embedded peptides and cell-associated fragments. This mechanism aligns with cheese ripening, where LAB autolysis contributes to peptide generation with antioxidant, antimicrobial, and ACE-inhibitory activity^98^. Kefir further exemplifies a complex postbiotic system in which EPS (kefiran) and microbial metabolites are linked to cardiometabolic and microbiome-modulating effects^99–101^.

Dairy products can also be fortified with defined postbiotic or paraprobiotic ingredients to improve robustness and dose consistency. Yogurt enriched with LAB-derived postbiotic powders showed increased antioxidant capacity and extended shelf life without compromising rheology properties^102^, while kefir grain powders improved water-holding capacity and texture without altering starter performance^103^. Heat-killed LAB can maintain yogurt physicochemical stability during refrigerated storage while avoiding viability-related issues such as post-acidification^104^. Commercial paraprobiotics such as heat-killed *L. paracasei* MCC1849 (LAC-Shield™) illustrate standardized dairy-compatible delivery systems, demonstrating stability through pasteurization and simulated gastric conditions together with immunomodulatory efficacy in humans^105^. Overall, fermented dairy matrices provide a scalable platform for postbiotics delivery, combining formulation robustness, controlled digestion-triggered release, and suitability for vulnerable populations^106^.

### Cereal-based products (bread, pasta, snacks)

Cereal-based foods impose major constraints on conventional probiotic delivery because baking, extrusion, and drying processes typically eliminate microbial viability. Paradoxically, these same processing stresses make cereals highly suitable matrices for postbiotics and paraprobiotics, since their functionality is not dependent on survival but rather on the persistence of fermentation-derived metabolites and structurally retained microbial components. Sourdough fermentation illustrates this concept: during proofing, LAB and yeasts generate organic acids, peptides, and EPS, whereas the subsequent baking step (>200 °C) converts the starter biomass into non-viable microbial structures and stabilizes a characteristic metabolite profile within the final bread^107^. Beyond processing stability, cereal matrices also influence gastrointestinal exposure. Starch gelatinization and protein network formation during baking can retain fermentation-derived compounds during storage, while digestion-driven matrix breakdown progressively releases soluble metabolites in the upper gastrointestinal tract. In parallel, fiber-associated fractions and bound fermentation products may reach the colon, where microbial metabolism can further shape their bioavailability and functional impact. This layered “processing-to-digestion” trajectory is particularly relevant for sourdough systems, where organic acids and antimicrobial peptides contribute to shelf-life extension while remaining compatible with postbiotic delivery^11,107^.

Targeted enrichment strategies further expand cereal functionality. Although probiotic incorporation into dough commonly results in near-complete loss of viability during baking^108^, microbial metabolic activity prior to inactivation can still enhance the postbiotic profile of the final product. For example, fermentation with *L. fermentum* increased γ-aminobutyric acid (GABA) levels in bread, supporting a neuroactive postbiotic signature despite subsequent heat inactivation^47^. Although probiotic incorporation into dough commonly results in near-complete viability loss during baking, microbial fermentation of gluten-free substrates can modify rheology and aroma through enzymatic transformation of cereal components and accumulation of organic acids and other metabolites that persist after processing^109^. Spore-forming bacteria represent an additional route for cereal fortification. *Bacillus* spp. may partially survive certain processes, yet studies indicate that both viable spores and the metabolites associated with spore germination dynamics can contribute to functional outcomes, supporting a paraprobiotic-compatible formulation strategy^110^. Encapsulation technologies further improves compatibility with high-temperature processing by protecting microbial structures and preserving intracellular-associated components even when survival is limited, effectively yielding microencapsulated paraprobiotics systems^111^. Similar concepts extend to pasta and extruded snacks products, where fermentation or inclusion of heat-killed preparations can generate products with enhanced antioxidant and immunomodulatory potential while remaining shelf-stable and industrially scalable^112^. Overall, cereals provide a robust platform for postbiotic-oriented product design, because the microbial “kill step” is intrinsic to processing, allowing formulation strategies to prioritize stable metabolites and inactivated microbial cells without viability constraints^21^.

### Beverages (functional drinks, plant-based alternatives)

Functional beverages are increasingly used as delivery platforms for postbiotics and paraprobiotics because matrices enable rapid formulation, homogeneous dispersion, and scalable thermal stabilization. Unlike live probiotics, postbiotic fractions generally tolerate pasteurization or ultra-high-temperature (UHT) processing, supporting stable products in which biological activity is decoupled from microbial viability^113,114^. From a matrix perspective, beverages differ from solid foods in that bioactive compounds are typically more readily bioaccessible, while instability is primarily driven by oxidation reactions, pH stress, and heat-sensitive molecules rather than structural entrapment. Experimental and formulation studies indicate that functional beverages fortified with postbiotic-rich fractions can maintain quality and biological activity through processing and storage, because postbiotic components remain stable and functionally active even after thermal treatments that inactivate live cells^16^. Similar approaches in fruit-based beverage systems show that fermentation followed by subsequent processing can generate postbiotic-rich drinks, in which organic acids and phenolic-associated antioxidant activity remain detectable after processing^115^.

These findings highlight a key advantage of beverage matrices, microbial metabolism can be exploited upstream during fermentation to enrich functional metabolites, while thermal treatment applied downstream ensures stability and product control. Fermented dairy drinks naturally contain organic acids, peptides, and EPS generated during lactic fermentation, and targeted fortification can further enhance standardized functional profiles. In yogurt-based smoothies, addition of postbiotic powders derived from *L. acidophilus* LA-5 improved bioactive retention during storage without impairing product quality or sensory properties^102^. Non-dairy beverages also provide strong opportunities, particularly because plant matrices contribute endogenous substrates such as β-glucans and polyphenols, which can be biotransformed during fermentation into more bioactive or bioaccessible forms. Comparable observations have been reported across cereal- and fruit-based beverages, where phenolic-associated activity remains sensorially acceptable after processing^116^. Beyond stability, beverage matrices may shape gastrointestinal exposure by enabling fast release of soluble postbiotic metabolites in the upper gastrointestinal tract, while microbial-derived cell fragments and polymeric compounds may persist longer and interact with mucosal surfaces depending on molecular size matrix composition, and formulation pH. Overall, functional beverages offer a scalable platform for postbiotic delivery, combining processing robustness, standardized dosing, and high bioaccessibility, with applicability across both dairy and plant-based product categories^16,114^.

### Encapsulation and delivery systems (spray-drying, micro/nanoencapsulation)

Encapsulation technologies are increasingly used to improve the stability, standardization, and gastrointestinal delivery of postbiotics and paraprobiotics in food systems. Although viability is not required, multiple postbiotic effectors remain vulnerable to thermal stress, oxygen exposure, pH fluctuations, and storage-driven chemical reactions. Encapsulation therefore functions as a formulation buffer that limits premature degradation while enabling controlled release kinetics across different food matrices^117^. Spray-drying remains the most scalable route to generate postbiotic and paraprobiotic powders, where fermented supernatants or inactivated biomass are co-processed with protective carriers such as maltodextrin, inulin, or dairy proteins. Properly optimized inlet and outlet temperature conditions can preserve key heat-tolerant markers and yield high retention of functional activity, supporting shelf-stable incorporation into beverages, fermented foods, and dry products^118^. However, retention is strongly molecule-dependent, as low-molecular-weight acids and volatile fractions may exhibit higher processing losses than polymeric or protein-associated bioactive compounds. Recent screening of technologically robust LAB isolates with antioxidant and stress tolerance traits further supports the selection of strains and fractions that better withstand downstream stabilization steps^119^. Beyond dehydration-based stabilization, hydrogel and emulsion encapsulation systems provide more precise control over gastrointestinal release. Alginate, carrageenan, and milk protein-based matrices can protect embedded bioactive fractions during gastric exposure and support pH-responsive or erosion-driven release in the intestine. In such systems, bioactivity is governed not only by chemical preservation but also by physical accessibility, as encapsulation may delay diffusion of peptides and structural fragments until intestinal conditions favor their liberation and interaction with mucosal surfaces^120^.

**Fig. 9:**
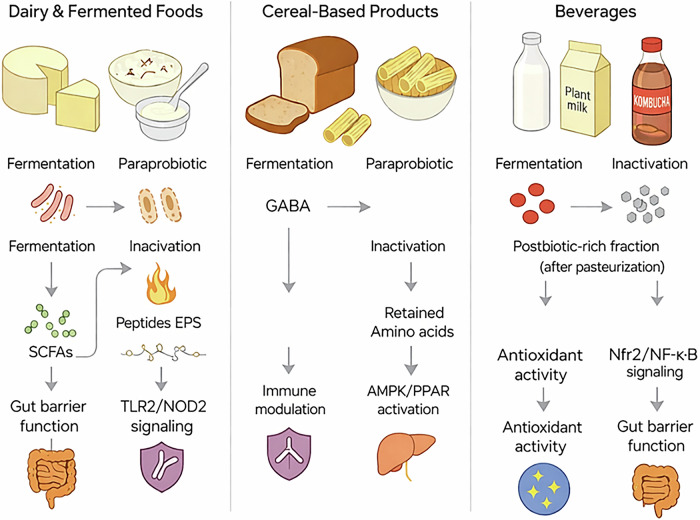
Food matrices as carriers of postbiotics and paraprobiotics. Dairy, cereal-based, and beverage matrices retain bioactive metabolites after fermentation and processing, providing health benefits such as support for the gut, immune system, antioxidant defense, and metabolism. The diagram illustrates how three primary food matrices (dairy, cereals, and beverages) retain bioactive metabolites following fermentation and inactivation processes. Downward arrows (↓) map the progression from these specific processing steps to their resulting physiological benefits, which include gut barrier support, immune modulation, metabolic activation, and antioxidant defense.

Emulsion-based carriers additionally enable co-delivery of hydrophilic and lipophilic bioactives, improving formulation compatibility and limiting partition-driven losses during storage^121^. Nanoencapsulation approaches further extend delivery control by improving resistance to bile salts, oxidative degradation, and pH-driven instability, while enabling delayed exposure of labile fractions through mucoadhesive behavior or particle-size–dependent gastrointestinal transit dynamics. Chitosan-based nanoparticles, polymeric nanolayers, and lipid nanocarriers have been explored to enhance retention of sensitive peptides and cell-associated bioactive structures, thereby supporting sustained interaction with epithelial and immune targets^122^. Such platforms may be particularly relevant when the intended postbiotic function depends on structure–function integrity, including surface motif recognition and receptor engagement. Accordingly, selecting strains and fractions with strong functional resilience remains a practical prerequisite for nano-enabled delivery strategies. From an application standpoint, encapsulation improves the feasibility of postbiotics in industrial foods by reducing sensory impact and ensuring dose consistency. Microcapsules in the ~50–100 µm range are frequently compatible with consumer acceptance in beverages and fermented systems, while the expanding use of generally recognized as safe (GRAS)-compatible wall materials supports scalable and sustainable encapsulation designs. Overall, encapsulation provides a critical technological bridge between postbiotic bioactivity and real-product performance by integrating molecular protection, matrix compatibility, and controlled gastrointestinal release into formulation pipelines (Fig. 9)^120,121^. Moreover, microalgae serve as supplementary sustainable bioactive platforms that exhibit molecular similarities with postbiotic therapies and provide novel delivery options for microbiome-targeted functional foods^123–125^.

### Processing stability (heat, pH, storage, shelf-life)

A major advantage of postbiotics and paraprobiotics over live probiotics is their superior stability during food processing and storage, since their functional potential is not constrained by microbial viability. This property provides substantial formulation flexibility, enabling their incorporation into baked, pasteurized, acidified, and shelf-stable products where live cultures would typically lose efficacy. Importantly, the persistence of postbiotic activity is molecule- and matrix-dependent, with different components exhibiting distinct sensitivities to thermal load, oxygen exposure, and physicochemical conditions. Thermal resilience is among the most consistently reported attributes of postbiotic preparations. Low–molecular weight metabolites such as lactic acid, acetic acid, and SCFAs, generally remain stable under conventional heat treatments, while structural constituents such as EPS and peptidoglycan-derived fragments often tolerate high processing temperatures because they are less susceptible to denaturation than viability-dependent microbial systems^16^.

Similarly, heat-stable bacteriocins, including nisin, can retain antimicrobial functionality after severe thermal exposure, supporting their integration into thermally processed food products^2^. Notably, many commercial paraprobiotic ingredients are intentionally produced through controlled heat inactivation processes, including tailored thermal treatments that selectively preserve structural and molecular fractions that remain functional after processing^126^. In parallel, encapsulation technologies and protective carriers systems can further enhance processing robustness, improving retention of heat- or oxidation-sensitive bioactive compounds in baked or extruded matrices^127^. Postbiotics also offer broad pH compatibility relative to live probiotics, allowing their use in acidic beverages and neutral food systems. Organic acids and cell envelope–associated components typically remain stable across moderate pH ranges, whereas peptide- and protein-based effectors may require protection against degradation or conformational destabilization, particularly under harsh gastric conditions. In such cases, delivery systems such as alginate–chitosan matrices can preserve functional integrity and improve site-specific gastrointestinal release^128^. Consistent with this concept, heat-killed *L. paracasei* MCC1849 (LAC-Shield™) has been incorporated into beverages while maintaining immunomodulatory performance across diverse formulation environments^129^.

From an industrial perspective, the elimination of viability constraints improves dose consistency and shelf-life predictability. Postbiotics and paraprobiotics can be dried and stored with reduced reliance on cold-chain logistics, supporting distribution in settings where refrigeration infrastructure is limited. For example, heat-killed *L. plantarum* can be incorporated into fermented dairy systems while avoiding viability loss and uncontrolled post-acidification, which often complicate live probiotic formulations^104^. Moreover, postbiotic powders and fermentate-derived fractions have demonstrated sustained preservative performance including antifungal activity, across extended storage periods, although retention depends on moisture control and oxygen exposure^11,130^. Collectively, postbiotics and paraprobiotics align strongly with industrial processing requirements by providing stable, shelf-compatible bioactivity, thereby expanding the feasible product space for functional foods and nutraceutical applications^2^.

### Matrix interactions and release kinetics

The efficacy of postbiotics and paraprobiotics is strongly influenced by their interactions with the food matrix and by their release kinetics during gastrointestinal digestion. Unlike live probiotics, whose performance is largely limited by viability and colonization, postbiotic functionality depends on the preservation, bioaccessibility, and site-specific liberation of microbial-derived effectors, making the matrix an active determinant of delivery rather than a neutral carrier^11^. Postbiotic components can undergo protein binding, lipid partitioning, or polysaccharide entrapment, processes that may simultaneously enhance stability and delay bioaccessibility. For example, EPS can integrate into gel or fiber networks, whereas peptide fractions may remain immobilized within protein-rich matrices until intestinal proteolysis occurs, enabling their release^131^. This phenomenon is particularly relevant in ripened cheeses, where fermentation-derived peptides remain matrix-bound during storage but become bioaccessible during digestion, thereby enabling delayed and potentially more targeted bioactivity^98^.

Similarly, in sourdough breads, inactivated LAB structures and fermentation metabolites can be retained within starch–gluten networks and progressively liberated during mastication and digestion^107^. However, matrix interactions can also attenuate activity when binding becomes excessive or when physicochemical incompatibilities limit diffusion. Acidic fermentates, for instance, may promote protein aggregation or induce viscosity shifts in high-protein systems, reducing the immediate availability of soluble postbiotic fractions and highlighting the need for matrix-specific optimization^48^. Release dynamics are equally decisive, liquid matrices typically favor rapid gastric liberation, whereas semi-solid and solid foods tend to promote delayed or multiphasic release profiles, resulting in prolonged exposure across the intestinal tract^132^. Encapsulation technologies further refine these kinetics by protecting labile compounds under gastric conditions and enabling controlled intestinal or colonic release, particularly through pH-responsive carriers such as alginate, chitosan, and enteric coatings^133,134^.

Notably, some food structures act as “natural encapsulants,” with protein-dense or fiber-rich matrices delaying release until enzymatic degradation or microbial fermentation occurs in distal compartments^135,136^. Matrix-mediated release can be extended by microbiota-driven secondary transformations. Exopolysaccharide and peptide complexes may act as fermentable substrates, supporting gradual bioconversion and sustained metabolic signaling, thereby linking matrix structure to postbiotic persistence and functional duration^131^. Overall, aligning matrix composition, molecular interactions, and digestion-responsive release profiles is essential for the rational design of predictable, standardized, and industrially compatible postbiotic foods^11^.

## Postbiotics in human nutrition

Postbiotics refer to preparations of non-viable microorganisms and/or their components that confer a health benefit to the host, encompassing inactivated cells, cell fragments, and bioactive metabolites that remain functional despite the absence of microbial replication^2,40,41^. Their increasing relevance in human nutrition largely stems from two translational advantages over live probiotics, improved formulation robustness (thermal/pH/storage tolerance) and reduced safety concerns in contexts where microbial viability is undesirable or contraindicated, particularly in vulnerable populations^76^. Current evidence supports postbiotic efficacy most consistently for gastrointestinal outcomes, while metabolic applications remain promising but are more variable, reflecting differences in postbiotic composition, dosing, host baseline phenotype, and microbiota context.

### Gastrointestinal health

**Fig. 10:**
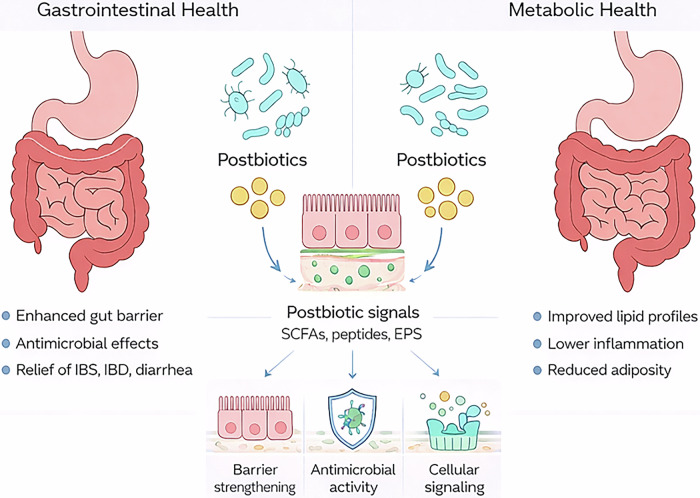
Postbiotic mechanisms supporting gastrointestinal and metabolic health. The diagram illustrates how postbiotic signals (SCFAs, peptides, and EPS) act on the intestinal epithelium to drive systemic benefits. The left side outlines gastrointestinal outcomes, such as enhanced barrier function and disease relief, while the right-side highlights metabolic improvements, including optimized lipid profiles and reduced adiposity.

Postbiotics have been explored across a spectrum of gastrointestinal conditions, including acute diarrhea, irritable bowel syndrome (IBS), and inflammatory bowel disease (IBD), with efficacy primarily mediated through barrier reinforcement, microbial ecosystem modulation, and immunometabolic signaling (Fig. 10). Mechanistically, fermentative metabolites support epithelial integrity by enhancing tight junction organization and mucus-associated defenses, while simultaneously shaping luminal ecology through pH reduction and antagonism against enteropathogens^21,137^. Importantly, these mechanisms do not require microbial survival, which explains why several inactivated preparations retain clinical activity in settings where probiotics lose viability. Diarrhea represents the most established clinical application. Heat-inactivated *L. acidophilus* LB has a long history of use as an antidiarrheal agent, and controlled studies in pediatric gastroenteritis indicate that postbiotic adjuncts can shorten symptom duration and improve clinical recovery metrics when combined with standard rehydration therapy^138^.

Similarly, fermented formulas containing inactivated strains have been associated with reduced dehydration severity and a lower need for clinical escalation, supporting a clinically relevant benefit profile in acute pediatric settings^138^. For IBS, evidence suggests that symptom improvement is achievable even with non-viable preparations, reinforcing the concept that visceral outcomes can be modulated through barrier and immune–neural mechanisms without colonization. Reports indicate that heat-killed formulations may improve stool consistency and abdominal discomfort in diarrhea-predominant IBS, and some trials suggest comparable symptomatic benefit between viable strains and their inactivated counterparts^40^. However, across IBS studies, heterogeneity in endpoints, placebo responsiveness, and subtype stratification remains a limiting factor for firm conclusions. In IBD, postbiotics are best viewed as adjunctive candidates rather than standalone therapeutics. Reduced endogenous SCFA availability and barrier dysfunction provide a strong mechanistic rationale, particularly for butyrate, which supports colonocyte bioenergetics and maintains anti-inflammatory immune responses^11^.

Preclinical studies show that culture supernatants can mitigate mucosal injury and shift microbial metabolic outputs in colitis models, sometimes approaching the performance of live-probiotic performance^139^. Early clinical signals from butyrate-based interventions are supportive but remain insufficient for definitive efficacy claims, underscoring the need for standardized formulations and adequately powered human trials^140^. Finally, constipation-related benefits remain mechanistically plausible but clinically underdeveloped. SCFAs can modulate motility-related physiology through smooth muscle contractility and secretion dynamics, and metabolite-driven effects on enteric signaling have been proposed^141^. Nevertheless, evidence is still limited and should be framed cautiously as an emerging direction rather than a validated application.

### Metabolic health

Metabolic applications represent a major frontier for postbiotic nutrition, supported by a compelling mechanistic framework linking microbial-derived effectors to host energy homeostasis and inflammation (Fig. 10). Key pathways include barrier tightening (reducing metabolic endotoxemia), activation of enteroendocrine signaling via free fatty acid receptor 2/3 (FFAR2/3), epigenetic modulation thorough HDAC inhibition, and downstream regulation of AMPK and PPAR-driven lipid oxidation programs^142^. Collectively, these mechanisms converge on improved insulin sensitivity, reduced hepatic lipid accumulation, and attenuation of chronic low-grade inflammation. Animal models provide strong mechanistic evidence. For example, MDP improves glucose tolerance through nucleotide-binding oligomerization domain-containing protein 2 (NOD2)-dependent immunometabolic signaling, reducing adipose inflammation independently of microbial replication^56^.

Other microbially derived metabolites have been associated with improved mitochondrial efficiency and metabolic phenotype correction in obesity-related models^143^. However, translation from rodents to humans remains non-trivial due to interindividual variability and differences in baseline microbiota–diet contexts. Clinical evidence is growing but remains mixed. Trials using butyrate-based interventions report improvements in hepatic steatosis-related indices, lipid profiles, and selected anthropometric outcomes in some cohorts, including pediatric obesity settings^144,145^. Yet, short-term interventions do not consistently yield robust glycemic or insulin-resistance improvements across studies, suggesting that postbiotic efficacy may depend on dosage, delivery kinetics, baseline metabolic phenotype, and responder stratification^79^. Notably, combining postbiotics with supportive substrates (synbiotic-like strategies) may offer a more durable metabolic impact by sustaining fermentation-linked signaling beyond the administered fraction^137^.

### Evidence gaps and priorities for translation

Despite strong mechanistic plausibility, postbiotic clinical translation is constrained by insufficient standardization and comparability across studies. “Postbiotic” remains an umbrella category that includes fundamentally different entities (metabolite-rich supernatants, purified molecules, inactivated intact cells, and fragmented structures), making cross-study synthesis difficult^40^. Future trials should prioritize chemically defined reporting (composition fingerprints), validated dose metrics (per molecule class), harmonized clinical endpoints, and host stratification (baseline microbiota and phenotype). Addressing these priorities will be essential to shift postbiotics from promising functional concepts to predictable, reproducible nutritional interventions with claim-ready evidence.

### Immunomodulation and infection resistance

Postbiotics can enhance infection resistance while promoting immune balance. At mucosal surfaces, SCFAs increase mucus secretion and antimicrobial peptide production, reinforcing the epithelial barrier against pathogen invasion and limiting inflammatory tissue damage^146^. Butyrate in particular supports tight-junction maintenance and epithelial resilience, reducing pathogen penetration and downstream immune hyperactivation^147^. At the immune-cell level, SCFAs signal through FFAR2/3 on neutrophils, macrophages, and dendritic cells, shaping cytokine outputs toward controlled inflammation while maintaining antimicrobial capacity^148^. In addition to metabolites, cell-wall structures such as peptidoglycan fragments and LTAs function as immune training cues via PRRs (TLRs, NOD-like receptors), supporting innate immune preparedness^149^. Clinically, bacterial lysates represent a validated postbiotic-like strategy: pediatric studies report reduced recurrent respiratory infections, consistent with enhanced mucosal immunity and sIgA responses^150,151^. A particularly relevant example is heat-killed *L. lactis* JCM 5805 (LC-Plasma), which activates plasmacytoid dendritic cells and promotes antiviral responsiveness; trials in stressed adults reported reduced respiratory symptom burden and increased antiviral immune markers^152^. Collectively, these data support postbiotics as immunonutrition tools capable of strengthening defense without viability-associated risks.

### Allergy prevention and modulation

Postbiotics provide a mechanistically attractive approach for allergy modulation, particularly in early life when immune education is highly sensitive to microbial cues. SCFAs such as butyrate promote regulatory T-cell differentiation and anti-inflammatory cytokine production (IL-10), favoring immune tolerance and reducing Th2-biased immune responses characteristic of allergic disease^153,154^. By improving epithelial barrier integrity, postbiotics can also reduce antigen translocation across the intestinal mucosa and subsequent immune sensitization to dietary proteins^137^. Evidence in pediatrics populations suggests that postbiotic-containing formulas and bacterial lysates preparations may contribute to reduced wheezing and fewer asthma exacerbations, likely through a combination of infection prevention and immune pathway modulation^155^. In infants with cow’s milk allergy, postbiotic-supplemented nutritional strategies have been associated with improved acquisition of immune tolerance, although outcomes remain formulation-dependent and require larger confirmatory clinical trials^126,156^. Overall, postbiotics appear particularly relevant in allergy-prone populations because they retain immune training potential while minimizing safety concerns associated with live microbial administration.

### Cognitive and neuropsychological effects

The gut–brain axis is a bidirectional signaling network linking intestinal microbial activity to central nervous system (CNS) regulation, and postbiotics are increasingly recognized as key mediators of this cross-talk. Unlike live probiotics, postbiotics (inactivated cells, cellular fragments, and/or microbial metabolites) can influence brain-related outcomes without requiring microbial survival or colonization, acting instead through integrated neural, endocrine, immune, and metabolic pathways^157^. This is particularly relevant for functional nutrition strategies targeting stress, mood, and cognitive resilience via stable microbial-derived bioactive compounds. Among postbiotic candidates, SCFAs are central due to their pleiotropic neurobiological actions. Butyrate, in particular, can modulate gene expression via HDAC inhibition and has been linked to reinforcement of blood–brain barrier integrity, reduced neuroinflammatory signaling, and improved tight-junction stability in preclinical models^157^. In parallel, SCFA-driven immunomodulation may shape microglial maturation and inflammatory tone, while gut–vagus signaling and enteroendocrine responses provide additional pathways linking intestinal metabolites to CNS circuits involved in stress and mood regulation^158^. Preclinical evidence supports meaningful psychotropic and neuroprotective potential, as sodium butyrate supplementation has demonstrated antidepressant-like effects, increased BDNF expression, and improved learning and memory performance in stress and aging models^159,160^. SCFAs have also been investigated in neurodegeneration-related pathways, including inflammation- and amyloid-related mechanisms^161^. Human evidence remains promising but still limited, with early trials and meta-analytic findings suggesting beneficial effects on depressive symptoms and stress-related outcomes; however, standardized postbiotic formulations and biomarker-driven clinical studies are still required to confirm causality and define optimal dosing strategies^157^.

### Clinical trials and meta-analyses: current evidence and gaps

The therapeutic relevance of postbiotics is increasingly supported by clinical trials conducted across heterogeneous populations, although the field remains in a consolidation phase with respect to standardization, cross-study comparability, and regulatory positioning. Human studies have explored postbiotics for gastrointestinal disorders, infection prevention, and metabolic dysfunction, with multiple randomized controlled trials (RCTs) reporting clinically meaningful benefits (Table 6). Evidence includes improved clinical outcomes in acute pediatric diarrhea, symptom reduction in diarrhea-predominant irritable bowel syndrome (IBS-D), and improvements in metabolic syndrome and non-alcoholic fatty liver disease (NAFLD)-related endpoints, alongside protective effects in pediatric respiratory infections^138,145,162^. A consistent translational advantage is enhanced safety and processing stability, making postbiotics particularly attractive for infants, older adults, and immunocompromised individuals, where live probiotic use may be restricted^40^. Notably, heat-killed *L. acidophilus* LB has been used as an adjunct antidiarrheal strategy without infection risk, including in pediatric and other vulnerable populations. Randomized and observational clinical studies indicate that inactivated microbial preparations can shorten the duration of acute diarrhea and improve recovery when administered alongside standard oral rehydration therapy, while maintaining an excellent safety profile^138^. Beyond diarrhea, controlled human trials and systematic syntheses reviews demonstrate that non-viable microbial preparations can retain probiotic-like efficacy across multiple indications. In IBS-D, randomized, double-blind trials report significant improvements in global symptom scores and stool consistency following administration of heat-treated microbial preparations, with effects comparable to those observed for the corresponding viable strains^163^. Clinical relevance has also been reported in extra-intestinal indications. In pediatric populations, postbiotic or bacterial lysate-based interventions have been associated with reduced incidence and severity of recurrent respiratory tract infections, consistent with enhanced mucosal immune responses and improved host resistance to infection^61^. In adults, postbiotic supplementation has demonstrated beneficial effects on bowel habits and symptom burden in chronic diarrhea, further supporting clinical activity independent of microbial viability^164^.

**Table 6:** Clinical and preclinical evidence of postbiotics in human nutrition

Domain	Study Design	Population/Model	Intervention	Outcomes and Results	Citation
Gastrointestinal Health	Systematic review of RCTs	Children with acute diarrhea	Heat-killed *Lactobacillus* LB + ORT	Consistent reduction in diarrhea duration and severity across pediatric RCTs	^138^
	Double-blind RCT	Adults with IBS-D (*n* = 120)	Heat-treated postbiotic ES1 (8 wk)	↓ IBS-SSS score, ↓ stool frequency, improved QoL vs placebo	^163^
	Double-blind RCT	Adults with functional GI symptoms (*n* = 96)	Yeast-derived postbiotic (12 wk)	↓ bloating, ↓ abdominal pain, improved stool form	^164^
Metabolic Health	Meta-analysis of RCTs	Adults with metabolic syndrome	Mixed postbiotics (SCFAs, inactivated LAB	↓ TG, ↓ HOMA-IR, ↓ waist circumference	^162^
	Animal (mice)	Diet-induced obesity	MDP		^196^
	Animal (mice)	High-fat diet	Postbiotic EPS from *Lactobacillus*		^247^
Immunomodulation	Position paper (clinical synthesis)	Pediatric populations	Heat-killed LAB & bacterial lysates	↓ respiratory infections; ↑ sIgA; excellent safety	^138^
	Double-blind RCT	Healthy adults (*n* = 241)	Heat-killed *L. paracasei* MCC1849	Maintained immune homeostasis; ↓ URTI incidence	^248^
	Double-blind RCT	Athletes	Heat-killed *L. lactis* JCM5805	↑ pDC activation; ↓ URTI symptoms	^152^
Allergy Modulation	Systematic review	Pediatric allergy & asthma	Postbiotics & bacterial lysates	↓ wheezing; ↓ asthma exacerbations (heterogeneous)	^155^
	Prospective cohort	Infants with CMA	Synbiotic/postbiotic-enriched formula	↑ immune tolerance acquisition (formulation-dependent)	^156^
Cognitive Health	Animal (mice)	Stress-induced depression	Heat-killed LAB	↓ anxiety- & depression-like behavior; ↑ BDNF	^160^
	Animal (mice)	Cognitive impairment	Heat-killed *Leuconostoc* H40	↑ memory; ↓ neuroinflammation; ↑ BDNF	^249^
	Review (mechanistic)	Preclinical CNS models	SCFAs & postbiotics	Neuroprotection via HDAC inhibition & immune signaling	^159^

A consistent translational advantage emerging across these studies is the favorable safety profile and formulation stability of postbiotics. Adverse events are generally comparable across postbiotic, probiotic, and placebo groups, and no infection-related risks have been reported. Collectively, current clinical evidence supports postbiotics as a promising nutrition-based strategy capable of delivering probiotic-like benefits while offering improved safety margins and manufacturing robustness. However, broader implementation and evidence-based clinical recommendations will require further standardization of formulations, harmonization of clinical endpoints, and adequately powered multicenter RCTs.

## Applications of postbiotics in animal nutrition

In animal production, postbiotics are increasingly positioned as practical functional feed additives to support performance and health under modern farming constraints, particularly where antibiotic reduction strategies and probiotic instability remain significant challenges (Table 7). Their interest lies in their technological robustness during feed processing and storage, as well as in their capacity to deliver reproducible biological effects without depending on microbial survival. In broiler chickens, supplementation with encapsulated postbiotics has been shown to improve growth performance, carcass traits, immune parameters, and physiological markers of development^165^.

**Table 7:** Animal-based studies evaluating the biological effects of postbiotic molecules

Animal Species	Type of Postbiotic	Dosage	Main Outcomes	Summary of Results	Reference
Rabbit (broiler)	*E. mundtii* EM 41/3 Mundticin-like bacteriocin	50 µL/animal·d (25,600 AU/mL in water)	↑ Phagocytic (non-specific) immunity (*p* < 0.05); no oxidative stress or negative blood effects; ↑ growth parameters and gut enzymatic (hydrolytic) activity	MLS EM 41/3 postbiotic significantly boosted innate immunity (phagocytosis) and growth in broiler rabbits without harming gut microbiota or meat quality.	^250^
Rabbit (male)	Fermented feed (*Lactobacillus*/yeast) *postbiotic*	2.0 kg/ton feed (0.2%)	↓ Abnormal sperm (30% → 22%) and tail defects; ↑ acrosome integrity; ↓ ALT and ALP (improved liver enzymes)	A lactic acid bacterium–based postbiotic in feed improved male rabbit semen quality (fewer abnormal sperm, better acrosomes) and liver function.	^251^
Rabbit (does, heat-stress)	Palm-date seed fermentation (*B. subtilis* + *S. cerevisiae*)	0.9 g/kg feed (free or nano-encapsulated)	↑ Feed intake; ↓ rectal temp and respiratory rate; improved hematological, immunological and antioxidant parameters; ↑ progesterone, litter size and weight	Supplementing heat-stressed rabbit does with this *Bacillus*/yeast postbiotic (especially nanoencapsulated) enhanced heat tolerance, immune/redox status and reproductive performance (higher litter size/weight).	^192^
Broiler chicken (NE challenge)	*L. acidophilus* fermentate (“Culbac” postbiotic)	1 kg/ton (starter), 0.5 kg/ton (grower/finisher) feed + 4 mL/L water	↓ NE lesion scores and mortality (postbiotic both feed+water: 16% vs 36% in untreated); ↑ weight gain, improved FCR; best results when combined with amoxicillin; outperformed probiotic alone	Culbac postbiotic dramatically controlled *C. perfringens*–induced necrotic enteritis: it lowered mortality and lesions and improved FCR and growth. It matched or surpassed probiotic treatment and was highly synergistic with antibiotic (10% mortality with Culbac+amoxicillin).	^205^
Rainbow trout (*Oncorhynchus mykiss*)	*Weissella cibaria*-derived postbiotic	0.50% of diet	↑ Gut LAB; ↑ IL-1β; ↓ IL-10, IL-8, IFN-γ, TNF-α expression; ~20.7% higher survival after *Yersinia ruckeri* challenge	A *W. cibaria* postbiotic modulated trout mucosal immunity (upregulating pro-inflammatory IL-1β, downregulating anti-inflammatory IL-10/IL-8/TNF) and enriched gut LAB, leading to significantly enhanced resistance to bacterial (Y. ruckeri) infection.	^252^
Pacific white shrimp (*Penaeus vannamei*)	SCFPs, “DVAQUA”	0.35% of diet	↑ Weight gain, SGR, protein/lipid efficiency; ↑ intestinal protease and hepatopancreas amylase; no change in body composition; ↑ disease resistance to *Vibrio parahaemolyticus* (lower mortality in challenge)	Feeding 0.35% SCFPs postbiotic significantly improved shrimp growth rates and feed efficiency, boosted digestive enzyme activities, and markedly reduced mortality under Vibrio challenge, thus enhancing innate immunity and potentially reducing antibiotic need.	^177^
Dairy cow (transition)	Mixed *Lactobacillus* fermentation products (postbiotic blends)	Supplementation during late gestation & early lactation (per study)	↑ DMI and digestibility; ↑ colostral IgG concentration; ↑ milk yield (especially fat/protein) and lactation persistency	Postbiotic supplementation during the transition period increased cows’ feed intake, nutrient digestibility and colostrum immunoglobulins, and led to higher milk production (notably fat and protein) and sustained lactation curves.	^181^
Dairy goat (lactating)	Yeast fermentation product (Probisan Ruminants)	3.75 g/goat·d	↑ Ruminal propionate; ↑ NDF/ADF digestibility (+~5%); ↑ fat-corrected milk yield ( + 0.48 kg/d) and milk protein/lactose; ↓ blood urea and ammonia; ↓ CH₄ emission per unit of milk fat/protein	Yeast postbiotic at 3.75 g/d enhanced fiber digestion and fermentation (higher ruminal propionate) in lactating goats, boosting milk yield and composition, and improving energy use (reduced methane per product).	^185^
Lamb (weaned) – gut & immunity	*L. plantarum* RG14 cell-free postbiotic	0.9% of diet	↑ Rumen papillae height/width; ↓ jejunal IgA (mucosal) and blood leukocytes; ↑ jejunal IL-6; ↓ IL-1β, IL-10, TNF; ↑ tight junction proteins (TJP-1, CLDN-1/4)	*L. plantarum* RG14 postbiotic (0.9%) enhanced rumen epithelial development and mucosal barrier function in lambs, and beneficially modulated immune gene expression (increased IL-6, downregulated IL-1β, TNF, IL-10).	^253^

**Fig. 11:**
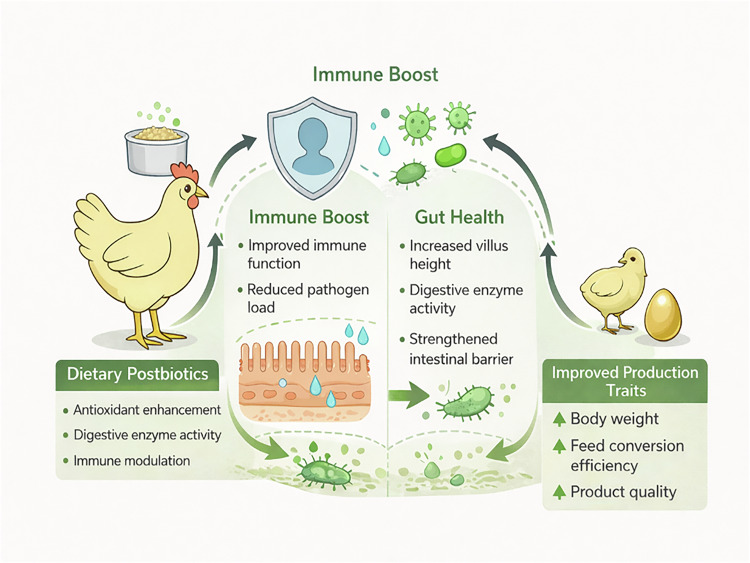
Postbiotic functions and advantages in poultry (immune stimulation, gut health, and production traits). The diagram illustrates how dietary postbiotics enhance poultry immune function and gut health, leading to improved production traits. Upward arrows (↑) denote increases in body weight, feed conversion efficiency, and product quality.

In poultry production, postbiotics have been linked to improvements in growth performance and feed efficiency, largely through coordinated effects on gut function and host defenses mechanisms. Reported outcomes include enhanced antioxidant status and immune responsiveness, alongside stabilization of intestinal microbiota composition and reduced pathogen pressure^165,166^. Figure 11 summarizes these core functional pathways in poultry, highlighting gut integrity reinforcement including improved villus morphology and digestive efficiency, immune stimulation, and microbiota modulation as central drivers of productive performance and product-quality-related benefits.

### Broiler chickens

In broilers, several studies report that postbiotics improve growth performance and feed efficiency. Atan Çırpıcı and Kırkpınar^165^ showed that broilers fed 0.3% encapsulated *Lactobacillus*-derived postbiotics achieved significantly higher final body weights and average daily gains compared with controls, with feed conversion ratio (FCR) notably improved during the starter phase. Carcass traits also improved through breast and thigh yields increased, abdominal fat deposition was reduced, and immune organ weights such as the bursa of Fabricius were higher, reflecting both improved growth performance and immune development. Similarly, postbiotic- and probiotic-based nutritional strategies have demonstrated efficacy in broilers exposed to production stressors, with performance benefits comparable to those observed in antibiotic-fed controls groups. In a controlled feeding trial, broilers supplemented with LAB isolated from fermented agro-industrial by-products showed significantly higher final body weight (up to 2787.5 g vs. 2356.8 g in controls, *p* < 0.05) and improved FCR, particularly in groups receiving *L. brevis* strains isolated from fermented green tea waste. These improvements were accompanied by enhanced meat quality parameters, including higher protein content (22.5% vs. 21.36%), increased intramuscular lipid content, and reduced lipid oxidation (TBARS: 0.59–0.60 vs. 0.82 mg MDA/kg in controls), indicating improved oxidative stability and metabolic efficiency^166^.

Sureshkumar et al.^167^ also reported that yeast cell wall–derived postbiotics enhanced feed efficiency in broilers by improving nutrient digestibility and energy metabolism, suggesting multiple postbiotic sources activate growth-related pathways. Postbiotics also markedly promote gut health in broilers. Birds receiving *Lactobacillus*-derived postbiotics showed increased villus height and villus:crypt ratio in the duodenum, jejunum, and ileum compared with both antibiotic- and amino acid–supplemented controls groups^165^. These morphological improvements were associated with enhanced nutrient absorption. The intestinal microbiota composition also shifted toward a more beneficial profile, with increased abundance of *Lactobacillus* spp. and reductions in potentially harmful bacteria such as *Salmonella* following yeast cell wall–derived postbiotic supplementation in broiler diets^168^. Furthermore, cecal pH was significantly lowered with postbiotic supplementation, reflecting increased lactic acid and SCFA production, which help suppress pathogens bacteria and support intestinal function^169^. Fermentation-derived feed ingredients and postbiotic-enriched diets have also been shown to improve intestinal health and microbial balance in broiler chickens. Recent evidence indicates that fermented soybean meal and other fermented feeds ingredients promote a more favorable gut microbiota composition, characterized by increased populations of lactic acid–producing bacteria and reduced abundance of potentially harmful microorganisms. These changes occur alongside improvements in intestinal morphology and barrier integrity, collectively supporting resistance to dysbiosis and enhanced nutrient utilization^170,171^.

These gut-level benefits are accompanied by improvements in immune function, as fermentation-derived metabolites and inactivated microbial components have been associated with enhanced humoral immune responses and modulation of growth- and immunity-related signaling pathways in broilers, particularly under conditions of nutritional or environmental stress^165^. Together, these findings support the role of postbiotics and fermentation-processed feed ingredients as functional nutritional strategies capable of improving gut health, immune competence, and overall resilience in modern broiler production systems. A key advantage of postbiotics in broiler production is their positive effect on meat quality and oxidative stability. Under heat-stress conditions, broilers receiving postbiotic supplementation exhibit enhanced antioxidant defenses, including increased activities of superoxide dismutase (SOD) and catalase, improved total antioxidant capacity, and reduced lipid peroxidation, as indicated by lower malondialdehyde (MDA) levels in plasma and muscle tissues. These changes indicate improved oxidative resilience under thermal stress^165,170^.

The antioxidant improvements induced by postbiotic supplementation translate into measurable enhancements in broiler meat quality. Recent studies report improved muscle oxidative stability, reflected by reduced lipid peroxidation, better preservation of muscle pH, enhanced water-holding capacity, and attenuation of excessive meat paleness. These combined effects collectively contributing to improved consumer acceptance and extended shelf stability^165,172^. In parallel, postbiotic supplementation has been associated with favorable modulation of lipid metabolism, including reductions in circulating lipid oxidation markers and improvements in systemic antioxidant–lipid balance, suggesting a link between postbiotic-driven redox regulation and lipid homeostasis^165^.

### Aquaculture species

#### Finfish

**Fig. 12:**
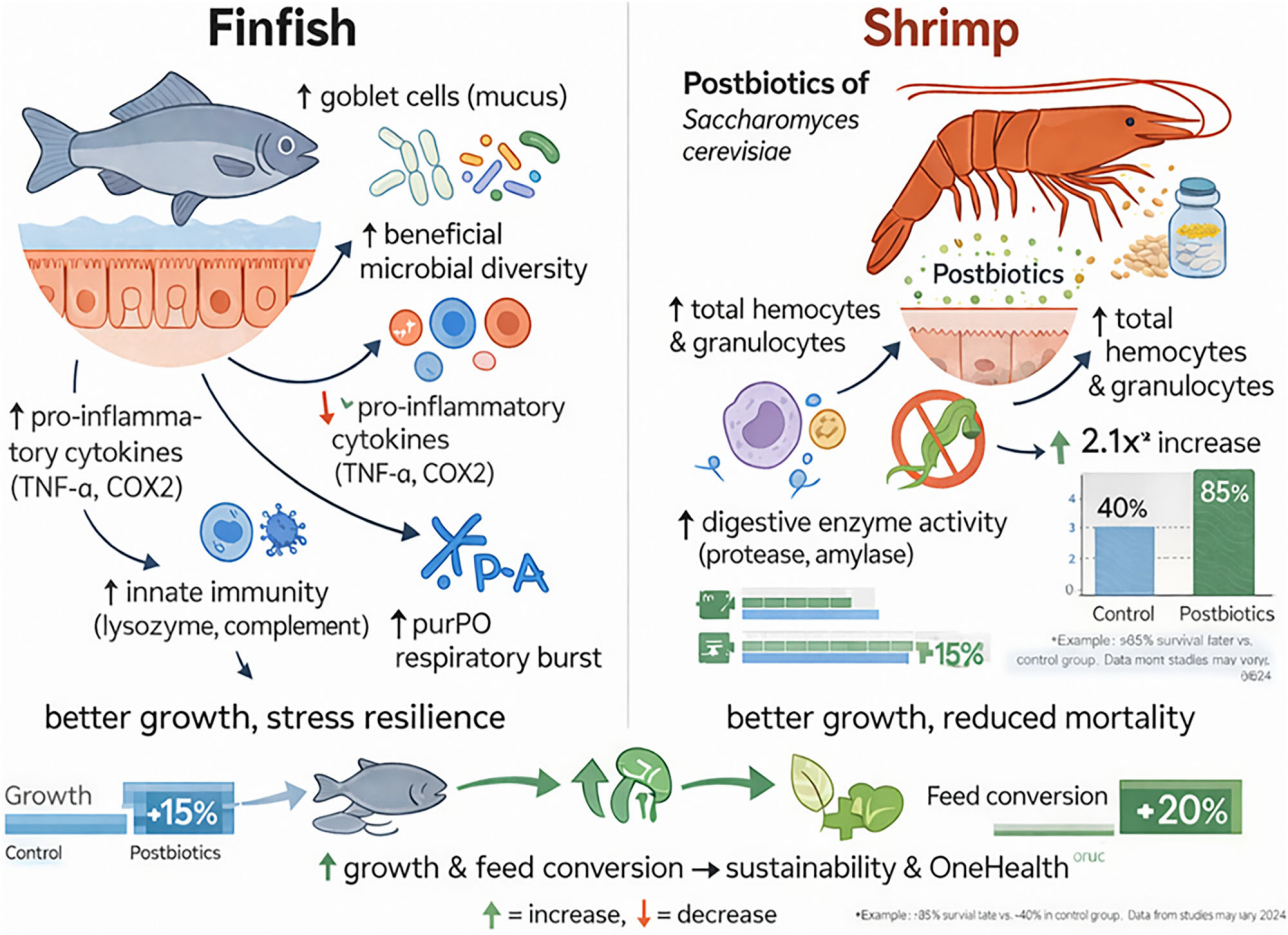
Beneficial roles of postbiotics in aquaculture. The diagram compares the physiological and immunological benefits of postbiotic application in finfish (left) and shrimp (right). In finfish, postbiotics enhance mucosal immunity and microbial diversity, while in shrimp, they boost hemocyte counts and digestive enzyme activity. Both pathways lead to improved growth, disease resistance, and feed conversion, supporting broader sustainability and One Health goals.

Recent studies have underscored the growing importance of postbiotics in aquaculture feed formulations to improve gut health, enhance immune competence, and increase stress resilience (Fig. 12). In gilthead seabream (*Sparus aurata*), supplementation with heat-inactivated *Vibrio proteolyticus* cultures has been shown to significantly improve gut barrier integrity and immune regulation. Cerezo et al^173^. demonstrated that this supplementation promoted a more diverse and balanced gut microbiota, with notable increases in beneficial genera, such as *Pseudomonas* and *Sphingomonas*, alongside reductions in potentially harmful taxa. Histological analysis revealed increased numbers of goblet cells in the intestinal epithelium and an upregulation of epithelial adhesion proteins including cadherin 1, *cdh1*, following a lipopolysaccharide challenge. Furthermore, basal levels of pro-inflammatory cytokines, including TNF-α and cyclooxygenase-2 (COX2), were downregulated, indicating a reduction in intestinal inflammation and enhanced epithelial integrity^173^.

In addition to Vibrio-derived postbiotics, extracellular products (ECPs) from *Shewanella putrefaciens* Pdp11 (SpPdp11) have been shown to exert immunoprotective effects in European seabass (*Dicentrarchus labrax*). In ex vivo assays, SpPdp11-ECPs reduced leukocyte apoptosis induced by *Photobacterium damselae* toxins while simultaneously enhancing phagocytosis activity and respiratory burst responses. These findings support the potential of SpPdp11-ECPs as non-cytotoxic immunomodulators that could help improve the fish’s immune responses under pathogen challenge^174^. Similarly, in tilapia (*Oreochromis niloticus*), postbiotics derived from *Saccharomyces cerevisiae* have been studied for their beneficial effects on growth performance, feed utilization, and immune responses. Ndambuki^175^ reported that dietary supplementation with yeast-derived postbiotics improved growth parameters, enhanced feed conversion efficiency, and promoted a favorable hematological profile in tilapia. Additionally, supplementation improved resistance to *Aeromonas veronii* challenge and enhanced the fish’s tolerance to fluctuating water quality conditions, resulting in fewer clinical signs of disease^175^.

#### Shrimp

In shrimp aquaculture, postbiotics have demonstrated substantial effects on immune function, survival rates, and overall health (Fig. 12). A prominent example is the use of *S. cerevisiae* fermentation products (SCFPs), which have been widely studied in whiteleg shrimp (*Penaeus vannamei*). Nguyen^176^ found that supplementation with SCFPs at a concentration of 0.45% resulted in significant improvements in several immune parameters, including total hemocyte count, granulocyte levels, prophenoloxidase activity, and respiratory burst responses. Additionally, when challenged with *Vibrio parahaemolyticus*, shrimp fed SCFPs exhibited a survival rate of 82.2%, representing a marked improvement compared to the 45.9% survival in control groups. Similarly, Prabu et al.^177^ evaluated the effects of SCFPs at varying inclusion levels (0%, 0.25%, 0.35%, and 0.45%) on shrimp health and performance. They found that a 0.35% inclusion level of SCFPs resulted in superior growth performance, including higher weight gain, specific growth rate, protein efficiency ratio, and lipid utilization, compared to the control group. Enhanced digestive enzyme activity, such as intestinal protease hepatopancreas amylase and protease activities, further supported the conclusion that SCFPs supplementation improves nutrient digestion and absorption efficiency. Notably, shrimp supplemented with SCFPs also exhibited significantly lower cumulative mortality following a *Vibrio* challenge across all SCFPs-fed groups. These findings align with recent literature demonstrating that postbiotics not only enhance growth performance and feed efficiency in aquaculture but also strengthen immune responses and reduce disease-related mortality. For example, dietary supplementation with *S. cerevisiae* fermentation-derived postbiotics significantly increased hemocyte responses and survival rates in *Litopenaeus vannamei* following bacterial challenge, underscoring their disease mitigation potential^178^. Broad reviews also highlight postbiotics’ capacity to improve gut health, growth performance, and pathogen resistance, positioning them as eco-friendly alternatives to antibiotics in sustainable shrimp farming systems^179,180^.

### Livestock

#### Ruminants

In dairy cows, postbiotics have been shown to improve feed intake, nutrient utilization, and milk production, particularly during the transition period around calving. Vicente et al.^181^ found that Holstein cows supplemented with a yeast-derived postbiotic during late gestation and early lactation exhibited higher dry matter intake (DMI), along with improved digestibility of dry matter (DM), organic matter (OM), and neutral detergent fiber (NDF). These improvements contributed to increased milk yield and improved milk composition, notably higher fat and protein content. Additionally, colostrum yield and quality were enhanced, with higher immunoglobulin G (IgG) levels, which strengthen passive immunity transfer to calves^181^. Furthermore, postbiotics have been shown to alleviate negative energy balance during early lactation by improving ruminal fermentation efficiency and enhancing energy extraction from feed. For example, supplementation with postbiotics during the transition period increased DMI and total-tract fiber digestibility, supporting improved energy utilization and higher milk yield in dairy cows^182,183^. Mechanistically, postbiotic supplementation has been associated with stabilization of rumen pH, increased volatile fatty acid production, and enrichment of fibrolytic microbial taxa^164,183^.

Postbiotic supplementation in transition dairy cows has been shown to exert antioxidant and immunomodulatory effects, including reductions in oxidative stress biomarkers and enhancements in immune function. Specifically, research indicates that supplementation with SCFPs during the periparturient period decreases plasma MDA concentrations and favorably modulates metabolic and immune parameters, thereby helping to protect cows from oxidative stress during calving and early lactation^181,184^. Additionally, postbiotics supplementation increased anti-inflammatory markers IL-10 and reduced pro-inflammatory cytokines (TNF-α, IL-6), supporting both cow health and productivity performance during early lactation^111^. In smaller ruminants such as sheep and goats, postbiotic yeast fermentation products increased ruminal propionate concentration and fiber digestibility while decreasing methane-related energy losses in lactating goats, thereby supporting improved energy efficiency and productive performance^185^. Additionally, recent study indicate that postbiotics improve volatile fatty acid profiles and stimulate fibrolytic microbial populations in the rumen. These changes may underlie enhanced feed efficiency and nutrient extraction in small ruminants^186^.

In beef cattle, postbiotics have been shown to improve feed efficiency and ruminal fermentation dynamics. Fernandez-Marenchino et al.^187^ reported that steers supplemented with 28 g/day of a SCFPs exhibited significantly higher total-tract digestibility of DM, OM, and starch compared to controls. This response was accompanied by an improved ruminal acetate-to-propionate ratio, indicating enhanced fermentation efficiency. Although methane production was not consistently reduced across trials, recent evidence indicates that supplementation with SCFPs can shift rumen fermentation toward propionate-producing pathways, thereby reducing hydrogen availability for methanogenesis and lowering methane yield under specific dietary and physiological conditions^188^.

Consistent antioxidant effects of postbiotics have been reported across ruminant species. Recent work in dairy cows demonstrated that supplementation with yeast-derived fermentation products enhanced systemic antioxidant defenses by increasing the activity of key enzymes such as SOD and glutathione peroxidase (GPx), while reducing lipid peroxidation markers during physiologically stressful periods^184^. Similarly, in young small ruminants, postbiotic supplementation has been shown to improve antioxidant biomarkers profiles and immune responsiveness, thereby contributing to healthier growth and increased resilience to environmental stressors^189,190^.

#### Rabbits

Postbiotics have shown considerable potential in improving growth performance, reproductive outcomes, and gut immunity in rabbits. Several studies have highlighted their effectiveness during key physiological stages, such as weaning and heat stress, by enhancing nutrient utilization, immune responses, and overall health status. In weaned rabbits, supplementing with the enterocin *Ent7420* has led to significant improvements in body weight gain and FCR. Pogány Simonová et al.^191^ demonstrated that *Ent7420* supplementation mitigated intestinal damage caused by the pathogen *Staphylococcus epidermidis*. This protective effect was achieved by preserving jejunal villus morphology and enhancing antioxidant enzyme activity, particularly serum GPx, which plays a vital role in reducing oxidative stress. Even in unchallenged rabbits, *Ent7420* improved GPx activity and phagocytic function, indicating systemic immunostimulatory effects that enhance overall immunity competence^191^.

In rabbits, postbiotic supplementation has demonstrated benefits beyond growth performance, including positive effects on reproductive health, antioxidant status, and immune function. In heat-stressed rabbit does, supplementation with a postbiotic derived from *Bacillus subtilis* and *S. cerevisiae*, particularly in nano-encapsulated form, increased feed intake, reduced physiological indicators of heat stress, enhanced reproductive outcomes such as kindling rate and litter size, and improved hematological, immunological, and redox parameters during pregnancy^192^. Additionally, postbiotic additives have also been shown to strengthen antioxidant defenses and immune responses in rabbits, increasing activities of enzymes like GPx and supporting gut barrier integrity, which contributes to overall health and resilience^191^.

## Comparative insights: postbiotics vs. probiotics vs. antibiotics

The comparative evaluation of postbiotics, probiotics, and antibiotics in animal nutrition highlights postbiotics as a new third pillar of feed supplementation that seeks to balances efficacy, safety, and technological robustness. Antibiotics have traditionally been used in animal production as growth promoters and disease preventatives. Their mechanisms involve broad-spectrum inhibition of bacterial protein synthesis, disruption of cell wall integrity, or DNA replication. However, long-term and subtherapeutic use has contributed to the emergence of AMR, which is now recognized as a major global public health concern^2^. In contrast, probiotics control pathogens through competitive exclusion, production of antimicrobial compounds such as bacteriocins, and modulation of host immune responses^193,194^. However, their effectiveness depends on maintaining sufficient viable populations in the gut, which can be compromised by feed processing, storage conditions, and gastrointestinal stresses such as gastric acidity and bile^195^. Postbiotics overcome many of the limitations associated with live probiotics by delivering preformed antimicrobial metabolites and structurally preserved microbial components that act independently of microbial viability. Organic acids produced during fermentation lower intestinal pH and suppress enteropathogens, while bacteriocins and antimicrobial metabolites such as reuterin exert direct inhibitory effects against *Clostridium perfringens* and *Salmonella*^17,196^. Recent poultry challenge studies demonstrate that *Lactobacillus*-derived postbiotics significantly reduce intestinal pathogen load, lesion severity, and mortality in necrotic enteritis models, highlighting their rapid and stability-driven antimicrobial activity without reliance on live bacterial colonization^170^. While antibiotic growth promoters historically improved FCR and body weight gain by suppressing subclinical infections and microbial competition for nutrients, postbiotics and probiotics enhance performance through improved digestion, microbiota modulation, and intestinal integrity, thereby offering a sustainable alternative to antibiotics^194^.

Postbiotics can deliver antimicrobial, gut-health, and performance benefits without the principal limitations associated with live probiotics, including viability loss during processing and storage and variability in intestinal colonization. Previous studies focused on poultry nutrition indicate that postbiotics encompassing cell-free metabolites, cell fragments, and inanimate microbial preparations can support growth performance and feed efficiency while improving intestinal function and immune balance, thereby positioning them as practical alternatives in antibiotic-reduction strategies^197^. In challenge models, postbiotic supplementation has demonstrated protective effects in broilers against necrotic enteritis, including reduced intestinal lesions and improved expression of nutrient transporter genes during recovery, findings consistent with enhanced gut resilience^198^. In addition, a postbiotic intervention evaluated with and without vaccination during an avian pathogenic *E. coli* challenge further supports the concept that postbiotics can contribute to disease mitigation in vaccinated broilers^199^.

Mechanistically, postbiotic antimicrobial activity is plausibly mediated by preformed metabolites and bioactive structural components. For example, reuterin-producing *L. reuteri* demonstrates inhibitory effects against pathogens, including *C. perfringens*, and reuterin has exhibited antimicrobial activity against *Salmonella*^200^. Additionally, bacteriocins such as plantaricins as antimicrobial peptides with pathogen-inhibitory potential. This selective yet potent activity strengthens the mechanistic basis for postbiotic approaches, as these bioactive metabolites can contribute to pathogen suppression without the need for administering viable bacterial cells^201^. From a safety and One Health perspective, recent studies highlight that live probiotics may pose risks in vulnerable hosts, including rare cases of bacteremia or sepsis and that AMR gene carriage and the potential horizontal gene transfer remain important safety considerations for probiotic products^3^. Because postbiotics are non-viable, they avoid risks associated with microbial replication and reduce concerns related to viability maintenance during processing and storage, while still delivering bioactive microbial-derived molecules with functional relevance^16^.

## Omics and AI-driven innovations in postbiotic research

### Standardized characterization of postbiotics

Postbiotics, the bioactive compounds derived from inactivated microorganisms, require clear and standardized frameworks for characterization to ensure scientific reproducibility and regulatory credibility. Key parameters for quality assurance include identity, potency, and stability, which are fundamental for both research applications and product development. The identity of postbiotics must be established through accurate taxonomic and genomic characterization of the parental strain used to generate the postbiotic preparation. Whole-genome sequencing (WGS) is considered the gold standard for confirming strain lineage, genetic integrity, and absence of undesirable traits such as transferable antimicrobial resistance determinants. This verification ensures that the bioactive properties attributed to a given postbiotic are reproducible across production batches and are unequivocally linked to the intended microorganism rather than to unintended microbial variation^2^. Furthermore, potency refers to the measurable bioactive content and functional capacity of the postbiotic preparation. Depending on the formulation, potency may be assessed by quantifying inactivated cell populations or by detecting and standardizing key bioactive molecules, including defined metabolites, peptides, or structural components. Analytical techniques like flow cytometry (FC), quantitative PCR (qPCR), and targeted chemical or biochemical assays are essential for ensuring that the postbiotic contains the necessary components for its expected health benefits. These methods support batch-to-batch consistency and facilitate mechanistic validation^20^. Additionally, stability represents another critical quality attribute. Although postbiotics are generally more stable than live probiotics, due to the absence of viability constraints, long-term efficacy still requires systematic stability evaluation. Postbiotic preparations are often resistant to environmental degradation and may be stored under ambient conditions, facilitating transport and formulation flexibility. However, stability-indicating assays and accelerated shelf-life testing are necessary to monitor bioactive integrity and prevent degradation-related loss of functional performance over time^10^.

### Artificial intelligence and omics approaches in discovering new bioactive molecules

The integration of omics technologies and artificial intelligence (AI) is revolutionizing the discovery and functional validation of novel bioactive molecules with postbiotic potential. These approaches combine top-down (phenotype-driven) and bottom-up (genome-mining and systems-biology–driven) strategies, offering a more comprehensive approach for identifying candidate bioactive compounds and elucidating their mechanisms of action. Genomic sequencing and metagenomic analyses are central to this discovery pipeline, as they enable identification of biosynthetic gene clusters responsible for the production of antimicrobial peptides, secondary metabolites, exopolysaccharides, and other structurally relevant molecules. By sequencing individual microbial genomes and analyzing complex microbial communities across diverse environments, researchers can identify strains with potential postbiotic-producing capabilities. This approach also includes in silico metabolite prediction, which utilizes existing genomic and biosynthetic gene cluster databases to identify promising molecules for experimental follow-up, helping to streamline the search for novel postbiotics^202^.

Metabolomics techniques, such as gas chromatography-mass spectrometry (GC–MS), liquid chromatography-mass spectrometry (LC–MS), and nuclear magnetic resonance (NMR), are instrumental in profiling the metabolites produced by probiotics strains. These techniques allow for the identification and quantification of various metabolites that contribute to the to the functional bioactivity of postbiotics. Metabolomic datasets provide a systematic catalog of postbiotic chemical diversity, which is crucial for identifying new bioactive molecules. As these databases expand and AI-assisted analytical pipelines become more sophisticated, the probability of discovering new postbiotic molecules with defined therapeutic potential correspondingly increases^203^. In addition, proteomics and secretomics, which involve the mass spectrometric analysis of extracellular proteins and peptides, provide another avenue for postbiotic discovery. These approaches enable identification of enzymes, antimicrobial peptides, structural proteins, and signaling molecules that contribute to postbiotic bioactivity. When integrated with genomic and metabolomic datasets, AI-driven multi-omics integration enables reconstruction of strain-specific metabolic networks, linking genes, proteins, and metabolites to functional host outcomes^202^. ML techniques, such as support vector machines (SVM) and random forests (RF) models, are used to analyze high-dimensional omics datasets. These methods can classify metabolites and peptides based on their predicted bioactivity profiles, identify molecular signatures associated with specific health outcomes, and detect hidden interaction patterns within complex datasets. As AI tools continue to evolve, they will significantly accelerate the discovery of new bioactive molecules and improve the efficiency of postbiotic production^204^.

### Personalized nutrition and microbiome stratification

Personalized nutrition, particularly in the context of postbiotics interventions, is emerging as a critical area of microbiome research. Interindividual variability in gut microbiome composition, genetics, dietary patterns, and immune status can strongly influence the physiological response to postbiotic supplementation. This variability underscores the need for personalized approaches to postbiotic therapy. Research into gut microbiome stratification is essential for understanding how individuals might respond to postbiotics. Just as humans are classified into different gut microbiome enterotypes, similar stratifications are possible in farm animals. For instance, poultry with distinct cecal enterotypes show varying microbiota compositions, which influence their susceptibility to pathogens and their response to postbiotic treatments. This suggests that categorizing individuals or animals based on their gut microbiota could optimize postbiotic therapies^20^. Beyond microbiome composition, host-specific factors such as genetics and immune system functionality also play key roles in determining how individuals respond to postbiotics. Identifying biomarkers that correlate with response to postbiotics, such as immune genotype or gut barrier integrity, is crucial for tailoring treatments. For example, genetic variants such as FUT2 secretor status can influence how individuals respond to bacterial cell-wall components, which may affect postbiotic efficacy^10^. The discovery of biomarkers predictive of postbiotic efficacy is vital for personalized nutrition. By analyzing metabolites and host markers including cytokines, and SCFAs before and after postbiotic supplementation, researchers can identify individuals who are most likely to benefit from specific postbiotic interventions. This approach moves beyond the traditional one-size-fits-all model and enables precision nutrition based on individual characteristics^20^.

### Synergistic multi-biotic strategies

The combination of multiple biotic components, including probiotics, prebiotics, and postbiotics, is gaining significant attention for its potential to enhance health outcomes. Multi-biotic strategies that synergistically integrate these components could provide more comprehensive benefits for gut health and overall well-being. Synbiotics, defined as the combination of probiotics and prebiotics, have long been shown to provide benefits beyond the effects of either component alone. Recent studies have demonstrated that synbiotics can improve gut health and microbial balance, often outperforming single-agent treatments. Similarly, combining postbiotics with prebiotics in synbiotic formulations may further optimize gut health by enhancing postbiotics activity while simultaneously supporting beneficial microbial growth. For example, combining a yeast probiotic with a prebiotic like mannan-oligosaccharide has been shown to significantly improve weight gain and reduce harmful bacteria in poultry^205^. Postbiotic-paraprobiotic combinations, which mix inactivated microbial cells with their bioactive metabolites, have also shown promising results. A recent study demonstrated that lozenges containing heat-killed *Lactobacillus* cells or cell-free supernatants from probiotics improved oral immunity and reduced pathogenic bacteria. This suggests that combining microbial cells with their associated metabolites may enhance therapeutic outcomes^202^. Looking beyond traditional combinations, the development of “superbiotics” which integrate probiotics, prebiotics, and postbiotics into a single formulation, represents a promising research direction. These multi-component products could provide a more holistic approach to gut health by promoting probiotic colonization while simultaneously delivering bioactive metabolites. The development of these superbiotics will require careful investigation of component compatibility, optimal dosing ratios, and underlying synergistic mechanisms o ensure safety, stability, and efficacy^10^.

### Sustainable postbiotic production and circular bioeconomy

Postbiotic production can contribute to sustainability goals by valorizing agro-industrial waste streams to generate high-value bioactive compounds. Utilizing food by-products as fermentation substrates aligns with circular bioeconomy principles by transforming low-value biomass into stable metabolites, including SCFAs, bacteriocins, and peptides^16^. Microbial fermentation of these residues using LAB and related cultures produces organic acids and other functional metabolites that can inhibit pathogens and promote gut health^45,206^. The resulting postbiotic-rich fermentates hold promise for incorporation into animal feed, where they may support beneficial gut microbiota and reduce pathogen loads, thereby further enhancing sustainability in animal production systems^207^. Continued development of cost-effective fermentation processes and improved valorization technologies will be key to the large-scale implementation of waste-derived postbiotics in both food and feed applications.

## Challenges, perspectives, and conclusions

Over the past decade, the field of functional foods has undergone a significant transformation, shifting from the traditional focus on probiotics to more stable and safer alternatives such as postbiotics and paraprobiotics. A growing body of evidence demonstrates that microbial-derived components can match or in some cases exceed the health benefits of live probiotics. This transition has important implications: it expands the range of microbiome-based treatments, improves product stability, and enhances safety for vulnerable groups, while also creating new opportunities for the development of food, nutraceutical, and pharmaceutical products. At the biochemical level, postbiotics include encompass a diverse array of molecules with multiple biological functions. SCFAs influence host metabolism and epigenetic processes; bacteriocins and antimicrobial peptides contribute to pathogen inhibition; EPS exhibit antioxidant and immune-modulating effects; and structural components such as MDPs and LTAs participate in regulate immune signaling pathways. These diverse mechanisms come together on key regulatory pathways, highlighting the therapeutic potential of postbiotics. Nevertheless, several challenges persist. Regulatory frameworks worldwide remain fragmented, and clear guidance regarding standardization, labeling, and health claim substantiation is still evolving. Although safety concerns, are generally favorable, thorough documentation remains essential, especially for applications in infant formulas, clinical nutrition, and pharmaceutical formulations. Methodological limitations also persist in areas such as accurate quantification of microbial derivatives, identification of strain-specific molecular signatures, and the establishment of reproducible analytical workflows for metabolites and proteins. These gaps emphasize the need for consensus-based frameworks, validated analytical assays, and harmonized guidelines to maintain quality control and consumer confidence.

Looking ahead, the integration of advanced technologies will reshape the future of postbiotic science. Multi-omics platforms including metabolomics, proteomics, and glycomics are expected to accelerate the discovery of new bioactive molecules, while ML and AI are poised to enhance biomarker discovery, stability prediction, and personalized nutrition strategies. Additionally, the combination of postbiotics, prebiotics, probiotics, and synbiotics may broaden the therapeutic possibilities through synergistic mechanisms. At the same time, sustainable bioprocessing and food-waste valorization approaches align postbiotics production with circular bioeconomy and One Health objectives. Crucially, translating these scientific advances into clinical applications and commercial products will require stronger collaboration among researchers, clinicians, industry stakeholders, and regulatory authorities. Postbiotics are now recognized as a distinct and promising category of functional ingredients, rather than just an extension of probiotics. They represent a convergence of microbiome science, food biochemistry, and clinical nutrition, offering a safe, stable, and sustainable approach for health promotion. Future progress will depend on integrating mechanistic insights with clearer regulatory frameworks, utilizing computational tools to accelerate discovery, and establishing postbiotics as key components of the next generation of functional foods and biotherapeutics.

## Data Availability

No datasets were generated or analyzed during the current study.
